# Intratympanic corticosteroids for Ménière’s disease

**DOI:** 10.1002/14651858.CD015245.pub2

**Published:** 2023-02-27

**Authors:** Katie E Webster, Ambrose Lee, Kevin Galbraith, Natasha A Harrington-Benton, Owen Judd, Diego Kaski, Otto R Maarsingh, Samuel MacKeith, Jaydip Ray, Vincent A Van Vugt, Brian Westerberg, Martin J Burton

**Affiliations:** Cochrane ENT, Nuffield Department of Surgical SciencesUniversity of OxfordOxfordUK; Department of Otolaryngology - Head and Neck SurgeryUniversity of TorontoTorontoCanada; Cochrane ENTNuffield Department of Surgical Sciences, University of OxfordOxfordUK; Ménière’s SocietyWootonUK; ENT DepartmentUniversity Hospitals of Derby and Burton NHS Foundation TrustDerbyUK; National Hospital for Neurology and NeurosurgeryLondonUK; Department of General Practice, Amsterdam UMCVrije Universiteit Amsterdam, Amsterdam Public Health Research InstituteAmsterdamNetherlands; ENT DepartmentOxford University Hospitals NHS Foundation TrustOxfordUK; University of SheffieldSheffieldUK; Otology & NeurotologySt. Paul's Rotary Hearing ClinicVancouverCanada; Cochrane UKOxfordUK

**Keywords:** Adult, Humans, Adrenal Cortex Hormones, Adrenal Cortex Hormones/adverse effects, Dexamethasone, Dexamethasone/adverse effects, Meniere Disease, Meniere Disease/complications, Meniere Disease/drug therapy, Tinnitus, Vertigo, Vertigo/drug therapy, Vertigo/etiology

## Abstract

**Background:**

Ménière's disease is a condition that causes recurrent episodes of vertigo, associated with hearing loss and tinnitus. Corticosteroids are sometimes administered directly into the middle ear to treat this condition (through the tympanic membrane). The underlying cause of Ménière's disease is unknown, as is the way in which this treatment may work. The efficacy of this intervention in preventing vertigo attacks, and their associated symptoms, is currently unclear.

**Objectives:**

To evaluate the benefits and harms of intratympanic corticosteroids versus placebo or no treatment in people with Ménière's disease.

**Search methods:**

The Cochrane ENT Information Specialist searched the Cochrane ENT Register; Central Register of Controlled Trials (CENTRAL); Ovid MEDLINE; Ovid Embase; Web of Science; ClinicalTrials.gov; ICTRP and additional sources for published and unpublished trials. The date of the search was 14 September 2022.

**Selection criteria:**

We included randomised controlled trials (RCTs) and quasi‐RCTs in adults with a diagnosis of Ménière's disease comparing intratympanic corticosteroids with either placebo or no treatment. We excluded studies with follow‐up of less than three months, or with a cross‐over design (unless data from the first phase of the study could be identified).

**Data collection and analysis:**

We used standard Cochrane methods. Our primary outcomes were: 1) improvement in vertigo (assessed as a dichotomous outcome ‐ improved or not improved), 2) change in vertigo (assessed as a continuous outcome, with a score on a numerical scale) and 3) serious adverse events. Our secondary outcomes were: 4) disease‐specific health‐related quality of life, 5) change in hearing, 6) change in tinnitus and 7) other adverse effects (including tympanic membrane perforation). We considered outcomes reported at three time points: 3 to < 6 months, 6 to ≤ 12 months and > 12 months. We used GRADE to assess the certainty of evidence for each outcome.

**Main results:**

We included 10 studies with a total of 952 participants. All studies used the corticosteroid dexamethasone, with doses ranging from approximately 2 mg to 12 mg.

**Improvement in vertigo**

Intratympanic corticosteroids may make little or no difference to the number of people who report an improvement in their vertigo at 6 to ≤ 12 months follow‐up (intratympanic corticosteroids 96.8%, placebo 96.6%, risk ratio (RR) 1.00, 95% confidence interval (CI) 0.92 to 1.10; 2 studies; 60 participants; low‐certainty evidence) or at more than 12 months follow‐up (intratympanic corticosteroids 100%, placebo 96.3%; RR 1.03, 95% CI 0.87 to 1.23; 2 studies; 58 participants; low‐certainty evidence). However, we note the large improvement in the placebo group for these trials, which causes challenges in interpreting these results.

**Change in vertigo**

***Assessed with a global score***

One study (44 participants) assessed the change in vertigo at 3 to < 6 months using a global score, which considered the frequency, duration and severity of vertigo. This is a single, small study and the certainty of the evidence was very low. We are unable to draw meaningful conclusions from the numerical results.

***Assessed by frequency of vertigo***

Three studies (304 participants) assessed the change in frequency of vertigo episodes at 3 to < 6 months. Intratympanic corticosteroids may slightly reduce the frequency of vertigo episodes. The proportion of days affected by vertigo was 0.05 lower (absolute difference ‐5%) in those receiving intratympanic corticosteroids (95% CI ‐0.07 to ‐0.02; 3 studies; 472 participants; low‐certainty evidence). This is equivalent to a difference of approximately 1.5 days fewer per month affected by vertigo in the corticosteroid group (with the control group having vertigo on approximately 2.5 to 3.5 days per month at the end of follow‐up, and those receiving corticosteroids having vertigo on approximately 1 to 2 days per month). However, this result should be interpreted with caution ‐ we are aware of unpublished data at this time point in which corticosteroids failed to show a benefit over placebo.

One study also assessed the change in frequency of vertigo at 6 to ≤ 12 months and > 12 months follow‐up. However, this is a single, small study and the certainty of the evidence was very low. Therefore, we are unable to draw meaningful conclusions from the numerical results.

**Serious adverse events**

Four studies reported this outcome. There may be little or no effect on the occurrence of serious adverse events with intratympanic corticosteroids, but the evidence is very uncertain (intratympanic corticosteroids 3.0%, placebo 4.4%; RR 0.64, 95% CI 0.22 to 1.85; 4 studies; 500 participants; very low‐certainty evidence).

**Authors' conclusions:**

The evidence for intratympanic corticosteroids in the treatment of Ménière's disease is uncertain. There are relatively few published RCTs, which all consider the same type of corticosteroid (dexamethasone). We also have concerns about publication bias in this area, with the identification of two large RCTs that remain unpublished. The evidence comparing intratympanic corticosteroids to placebo or no treatment is therefore all low‐ or very low‐certainty. This means that we have very low confidence that the effects reported are accurate estimates of the true effect of these interventions. Consensus on the appropriate outcomes to measure in studies of Ménière's disease is needed (i.e. a core outcome set) in order to guide future studies in this area, and enable meta‐analysis of the results. This must include appropriate consideration of the potential harms of treatment, as well as the benefits. Finally, we would also highlight the responsibility that trialists have to ensure results are available, regardless of the outcome of their study.

## Plain language summary

**What are the benefits and risks of corticosteroids given directly to the ear in Ménière's disease?**

**Key messages**

Due to a lack of robust evidence, it is not clear whether corticosteroids given into the ear (intratympanic corticosteroids) work to improve symptoms for people with Ménière's disease. It is also not clear whether there are any risks associated with treatment.

Larger, well‐conducted studies are needed in order to identify whether this treatment may be effective, and to assess whether there are any harmful effects.

Further work also needs to be done to find out how best to measure the symptoms of people with Ménière's disease, in order to assess whether treatments are beneficial or not. This should include the development of a 'core outcome set' ‐ a list of things that should be measured in all studies on Ménière's disease.

**What is Ménière's disease?**

Ménière's disease is a condition that affects the inner ear. It causes repeated attacks of dizziness or vertigo (a spinning sensation), together with hearing problems, tinnitus (ringing, humming or buzzing noises in the ears) and a feeling of fullness or pressure in the ear. It usually affects adults and starts in middle age.

**How is Ménière's disease treated?**

Oral medications (tablets) are often used as the first treatment for Ménière's disease. If these treatments do not control the symptoms, then corticosteroids may be given directly in the ear. This is most commonly given as an injection through the ear drum, but may be done by placing a grommet ‐ a tiny tube ‐ in the eardrum, and giving the corticosteroids as drops into the ear.

**What did we want to find out?**

We wanted to find out:

‐ whether there was evidence that intratympanic corticosteroids work at reducing the symptoms of Ménière's disease;

‐ whether this treatment might cause any serious harms, or other side effects (such as causing a hole in the eardrum).

**What did we do?**

We searched for studies that compared intratympanic corticosteroids to either no treatment or sham (placebo) treatment.

**What did we find?**

We found 10 studies, which included a total of 952 people. They lasted between three months and two years.

‐ When people considered whether their vertigo had improved, there was very little difference between those who had received intratympanic corticosteroids and those who had received no treatment (or sham treatment) at either six months to one year, or up to two years of follow‐up.

‐ When people counted the number of vertigo episodes they had, we found that intratympanic corticosteroids might reduce the number of episodes, but only by a small amount. This was the case when people were seen at three to six months, but we are not sure if the effect would also be seen at longer follow‐up times.

‐ It is unclear whether intratympanic corticosteroids increase the chance of experiencing serious medical problems (serious adverse effects).

**What are the limitations of the evidence?**

We have very little confidence in the evidence because most of the studies conducted were very small and had problems in their conduct, which means that the results may be unreliable. We also found two large studies that have not been published, therefore their results could not be included in this review. We understand that these studies found that intratympanic corticosteroids were not effective. If we had been able to include these data then some conclusions of this review might be different.

Larger, well‐conducted studies are needed to try and work out how effective the different treatments really are. In addition, the people conducting those studies must make sure that the results are available, regardless of the findings of the study.

**How up‐to‐date is this evidence?**

This evidence is up‐to‐date to September 2022.

## Summary of findings

**Summary of findings 1:** Intratympanic corticosteroids compared to placebo/no treatment for Ménière’s disease

**Intratympanic corticosteroids compared to placebo/no treatment for Ménière’s disease**						
**Patient or population:** adults with Ménière’s disease **Setting:** outpatient management **Intervention:** intratympanic corticosteroids **Comparison:** placebo or no treatment						
**Outcomes**	Anticipated absolute effects^*^ (95% CI)		**Relative effect (95% CI)**	**№ of participants (studies)**	**Certainty of the evidence (GRADE)**	**Comments**
	**Risk with placebo/no treatment**	**Risk with intratympanic corticosteroids**				
Improvement in vertigo frequency Assessed with: AAO‐HNS 1995 Class A, B or C Follow‐up: range 6 months to ≤ 12 months	Study population		RR 1.00 (0.92 to 1.10)	60 (2 RCTs)	⊕⊕⊝⊝ Low^1,2^	Intratympanic corticosteroids may have little or no effect on the number of people who experience an improvement in vertigo at 6 to ≤ 12 months.
	966 participants per 1000 would report that their vertigo had improved	966 participants per 1000 would report that their vertigo had improved (from 888 to 1000)				
Improvement in vertigo frequency Assessed with: AAO‐HNS Class A, B or C Follow‐up: range ≥ 12 months	Study population		RR 1.03 (0.87 to 1.23)	58 (2 RCTs)	⊕⊕⊝⊝ Low^1,2^	Intratympanic corticosteroids may have little or no effect on the number of people who experience an improvement in vertigo at ≥ 12 months.
	963 participants per 1000 would report that their vertigo had improved	992 participants per 1000 would report that their vertigo had improved (from 838 to 1000)				
Change in vertigo (global score) Assessed with: change from baseline in 'Gates Score' Scale from: 0 to 4, higher = worse Follow‐up: range 3 months to < 6 months	The mean change in vertigo (global score) was ‐0.467 points	MD 0.13 points lower (0.42 lower to 0.16 higher)	—	44 (1 RCT)	⊕⊝⊝⊝ Very low^2,3,4^	Intratympanic corticosteroids may have little or no effect on the change in vertigo at 3 to < 6 months, when measured using a global score of vertigo severity, frequency and duration.
Change in vertigo (frequency) Assessed with: change from baseline in proportion of days with definitive vertigo episodes Follow‐up: range 3 months to < 6 months	The mean change in vertigo frequency was ‐0.124 (the proportion of days affected by vertigo, equivalent to a reduction of about 3.8 days from baseline)	MD 0.05 (the proportion of days affected by vertigo) lower (0.07 lower to 0.02 lower)	—	472 (3 RCTs)	⊕⊕⊝⊝ Low^2,4^	Intratympanic corticosteroids may slightly reduce the frequency of vertigo episodes at 3 to < 6 months. This change would be equivalent to a reduction of about 1.5 days per month when compared to the control group (95% CI from 2.17 days to about 0.6 days per month fewer than the control group).
Change in vertigo (frequency) Assessed with: change in the number of episodes per month Follow‐up: range 6 months to ≤ 12 months	The mean change in vertigo frequency was ‐0.66 episodes per month	MD 0.1 episodes per month lower (0.79 lower to 0.59 higher)	—	20 (1 RCT)	⊕⊝⊝⊝ Very low^5,6^	The effect of intratympanic corticosteroids on vertigo frequency at 6 to ≤ 12 months is very uncertain.
Change in vertigo (frequency) Assessed with: change in the number of episodes per month Follow‐up: range > 12 months	The mean change in vertigo (frequency) was ‐0.77 episodes per month	MD 0.07 episodes per month lower (0.84 lower to 0.7 higher)	—	18 (1 RCT)	⊕⊝⊝⊝ Very low^5,6^	The effect of intratympanic corticosteroids on vertigo frequency at > 12 months is very uncertain.
Serious adverse events	Study population		RR 0.64 (0.22 to 1.85)	500 (4 RCTs)	⊕⊝⊝⊝ Very low^2,4,7^	The effect of intratympanic corticosteroids on serious adverse events is very uncertain.
	44 per 1000	28 per 1000 (10 to 82)				
*The risk in the intervention group (and its 95% confidence interval) is based on the assumed risk in the comparison group and the **relative effect** of the intervention (and its 95% CI). AAO‐HNS American Academy of Otolaryngology ‐ Head and Neck Surgery; **CI:** confidence interval; **MD:** mean difference; **RCT:** randomised controlled trial; **RR:** risk ratio						
**GRADE Working Group grades of evidence** **High certainty:** we are very confident that the true effect lies close to that of the estimate of the effect. **Moderate certainty:** we are moderately confident in the effect estimate: the true effect is likely to be close to the estimate of the effect, but there is a possibility that it is substantially different. **Low certainty:** our confidence in the effect estimate is limited: the true effect may be substantially different from the estimate of the effect. **Very low certainty:** we have very little confidence in the effect estimate: the true effect is likely to be substantially different from the estimate of effect.						

^1^High risk of performance and detection bias in one study, and attrition bias in the other study. ^2^Sample size fails to meet optimal information size (taken as < 400 participants for continuous outcomes, < 300 events for dichotomous outcomes). ^3^An unvalidated rating score was used to assess this outcome. ^4^We are aware of two unpublished trials from the same pharmaceutical company that apparently showed negative efficacy results. ^5^Risk of attrition bias. ^6^Extremely small sample size. ^7^Confidence interval ranges from potential benefit to potential harm.

## Background

### Description of the condition

Ménière's disease was first described by Prosper Menière in 1861 as a condition characterised by episodes of vertigo, associated with hearing loss and tinnitus (Baloh 2001). Sufferers may also report a feeling of fullness in the affected ear. Typically, it initially affects one ear, although some individuals may progress to develop bilateral disease. A hallmark of the condition is that symptoms are intermittent ‐ occurring as discrete attacks that last from minutes to several hours, then resolve. However, over time there is usually a gradual deterioration in hearing, and there may be progressive loss of balance function, leading to chronic dizziness or vertigo.

The diagnosis of Ménière's disease is challenging, due to the episodic nature of the condition, clinical heterogeneity and the lack of a 'gold standard' diagnostic test. Even the agreed, international classification system has scope for two categories of diagnosis – 'definite' and 'probable' (Lopez‐Escamez 2015). In brief, a diagnosis of definite Ménière's disease requires at least two episodes of vertigo, each lasting 20 minutes to 12 hours, together with audiometrically confirmed hearing loss and fluctuating aural symptoms (reduction in hearing, tinnitus or fullness) in the affected ear. 'Probable' Ménière's disease includes similar features, but without the requirement for audiometry to diagnose hearing loss, and with scope for the vertigo episodes to last longer (up to 24 hours). Both categories ('definite' and 'probable') require that the symptoms are not more likely to be due to an alternative diagnosis, due to the recognised challenges in distinguishing between balance disorders. 

Given the difficulties in diagnosis, the true incidence and prevalence of the disease are difficult to ascertain. A population‐based study in the UK using general practice data estimated the incidence to be 13.1 per 100,000 person‐years (Bruderer 2017), and the prevalence of the disease has been estimated at 190 per 100,000 people in the US (Harris 2010). It is a disorder of mid‐life, with diagnosis typically occurring between the ages of 30 and 60 (Harcourt 2014). Some studies report a slight female preponderance, and there may be a familial association, with approximately 10% of patients reporting the presence of the disease in a first, second or third degree relative (Requena 2014).

The underlying cause of Ménière's disease is usually unknown. Ménière's disease has been associated with an increase in the volume of fluid in the inner ear (endolymphatic hydrops). This may be caused by the abnormal production or resorption of endolymph (Hallpike 1938; Yamakawa 1938). However, it is not clear whether this is the underlying cause of the condition, or merely associated with the disease. Some authors have proposed other underlying causes for Ménière's disease, including viral infections (Gacek 2009), allergic (Banks 2012) or autoimmune disease processes (Greco 2012). A genetic predisposition has also been noted (Chiarella 2015). Occasionally, the symptoms may be secondary to a known cause (such as a head injury or other inner ear disorder) – in these cases it may be referred to as Ménière's syndrome.

Although Ménière's disease is relatively uncommon, it has a profound impact on quality of life. The unpredictable, episodic nature of the condition and severe, disabling attacks of vertigo cause a huge amount of distress. Quality of life (including physical and psychosocial aspects) is significantly reduced for those with Ménière's disease (Söderman 2002). The costs of the condition are also considerable, both in relation to medical interventions (appointments, diagnostic tests and treatments) and loss of productivity or sick days for those affected by the condition (Tyrrell 2016).

### Description of the intervention

A variety of different interventions have been proposed to treat people with Ménière's disease. These include dietary or lifestyle changes, oral treatments, treatments administered by injection into the ear (intratympanic) and surgical treatments. This review focuses on the use of intratympanic corticosteroids to treat the symptoms of Ménière's disease.

Corticosteroids can be administered into the middle ear through the tympanic membrane. They are often administered via injection, but can also be delivered as drops through a ventilation tube (with or without a wick). Treatment regimens vary from a one‐off injection to a short course (two to three injections), and may need to be repeated if symptoms recur. Different types of corticosteroids may be used, including methylprednisolone, dexamethasone or hydrocortisone. 

At present, there is no agreement on which is the ideal treatment for people with Ménière's disease – consequently there is no 'gold standard' treatment with which to compare these medications. 

### How the intervention might work

As the underlying cause of Ménière's disease is poorly understood, so too are the ways in which the interventions may work. 

The specific action of steroids in the inner ear is unclear, but may include an influence on water homeostasis, ion channels and blood flow to the inner ear (reviewed in Farhood 2016). The rationale for intratympanic delivery is that it enables steroids to reach the target organ at a high dose (Parnes 1999), whilst avoiding complications from systemic administration. The drug is thought to be absorbed into the inner ear, where glucocorticoid receptors have been shown to be present (Rarey 1996). 

Potential side effects of the intervention include pain due to the procedure, a persistent perforation of the tympanic membrane, or the development of tinnitus, vertigo or hearing loss following the injection. 

### Why it is important to do this review

Balance disorders can be difficult to diagnose and treat. There are few specific diagnostic tests, a variety of related disorders with similar symptoms, and a limited number of interventions that are known to be effective. To determine which topics within this area should be addressed with new or updated systematic reviews we conducted a scoping and prioritisation process, involving stakeholders (https://ent.cochrane.org/balance-disorders-ent). Ménière's disease was ranked as one of the highest priority topics during this process (along with vestibular migraine and persistent postural perceptual dizziness). 

Although Ménière's disease is a relatively uncommon condition, the significant impact it has on quality of life demonstrates the clear importance of identifying effective interventions to alleviate the symptoms. There is considerable variation in the management of Ménière's disease on both a national and international scale, with a lack of consensus about appropriate first‐line and subsequent therapies. 

This review is part of a suite of six that consider different interventions for Ménière's disease. Through these reviews, we hope to provide a thorough summary of the efficacy (benefits and harms) of the different treatment options, to support people with Ménière's disease (and healthcare professionals) when making decisions about their care. 

## Objectives

To evaluate the benefits and harms of intratympanic corticosteroids versus placebo or no treatment in people with Ménière's disease.

## Methods

### Criteria for considering studies for this review

#### Types of studies

We included randomised controlled trials (RCTs) and quasi‐randomised trials (where trials were designed as RCTs, but the sequence generation for allocation of treatment used methods such as alternate allocation, birth dates etc). 

Ménière's disease is known to fluctuate over time, which may mean that cross‐over trials are not an appropriate study design for this condition. No cross‐over RCTs or cluster‐RCTs were identified as relevant for inclusion in this review.

We included studies reported as full‐text, those published as conference abstracts only and unpublished data. 

Ménière's disease is characterised by episodic balance disturbance ‐ the frequency of attacks may change over time (Huppert 2010). For studies to obtain accurate estimates of the effect of different interventions, we considered that follow‐up of participants should be for at least three months ‐ to ensure that participants are likely to have experienced a number of attacks during the follow‐up period. Studies that followed up participants for fewer than three months were excluded from the review.

#### Types of participants

We included studies that recruited adult participants (aged 18 years or older) with a diagnosis of definite or probable Ménière's disease, according to the agreed criteria of the American Academy Otolaryngology ‐ Head and Neck Surgery (AAO‐HNS), the Japan Society for Equilibrium Research, the European Academy of Otology and Neurotology and the Bárány Society. These criteria are outlined in Appendix 1 and described in Lopez‐Escamez 2015. 

If studies used different criteria to diagnose Ménière's disease, we included them if those criteria were clearly analogous to those described in Lopez‐Escamez 2015. For example, studies that used earlier definitions of Ménière's disease (from the AAO‐HNS guidelines of 1995) were also included. If there was uncertainty over the criteria used for the study, then a decision was made on whether to include the study. This decision was taken by authors who were masked to other features of the studies (such as study size, other aspects of methodology, results of the study) to avoid the introduction of bias in study selection. If a study was conducted in an ENT department and participants were diagnosed with Ménière's disease then we considered it was likely that other diagnoses had been excluded and included the study. However, we reflected this uncertainty in diagnosis by considering the study at risk of indirectness when using GRADE to assess the certainty of the evidence (see 'Summary of findings and assessment of certainty of the evidence'). 

We anticipated that most studies would include participants with active Ménière's disease. We did not exclude studies if the frequency of attacks at baseline was not reported or was unclear, but we planned to highlight if there were differences between studies that may impact on our ability to pool the data, or affect the applicability of our findings.

We excluded studies where participants had previously undergone destructive/ablative treatment for Ménière's disease in the affected ear (such as vestibular neurectomy, chemical or surgical labyrinthectomy), as we considered that they were unlikely to respond to interventions in the same way as those who had not undergone such treatment.

#### Types of interventions

We included the following interventions:

- intratympanic corticosteroids:
  - including methylprednisolone, dexamethasone, hydrocortisone or other glucocorticoids.

The main comparison is:

- intratympanic corticosteroids versus placebo/no treatment.

We pooled all interventions, regardless of the type and concentration of steroid used, frequency and method of (intratympanic) delivery.  

##### Concurrent treatments

There were no limits on the type of concurrent treatments used, providing these were used equally in each arm of the study. We pooled studies that included concurrent treatments with those where participants did not receive concurrent treatment. We planned to conduct subgroup analysis to determine whether the effect estimates may be different in those receiving additional treatment. However, due to the small number of studies included in the review this was not possible (see Subgroup analysis and investigation of heterogeneity). 

#### Types of outcome measures

We assessed all outcomes at the following time points: 

- 3 to < 6 months;
- 6 to ≤ 12 months;
- > 12 months.

The exception was for adverse event data, when we used the longest time period of follow‐up. 

We searched the COMET database for existing core outcome sets of relevance to Ménière's disease and vertigo, but were unable to find any published core outcome sets. We therefore conducted a survey of individuals with experience of (or an interest in) balance disorders to help identify the outcomes that should be prioritised. This online survey was conducted with the support of the Ménière's Society and the Migraine Trust, and included 324 participants, who provided information regarding priority outcomes. The review author team used the results of this survey to inform the choice of outcome measures in this review. 

We analysed the following outcomes in the review, but did not use them as a basis for including or excluding studies.

##### Primary outcomes

- Improvement in vertigo
  - Measured as a dichotomous outcome (improved/not improved), according to self‐report, or according to a change of a specified score (as described by the study authors) on a vertigo rating scale.
- Change in vertigo
  - Measured as a continuous outcome, to identify the extent of change in vertigo symptoms.
- Serious adverse events
  - Including any event that causes death, is life‐threatening, requires hospitalisation, results in disability or permanent damage, or in congenital abnormality. Measured as the number of participants who experience at least one serious adverse event during the follow‐up period.

Vertigo symptoms comprise a variety of different features, including frequency of episodes, duration of episodes and severity/intensity of the episodes. Where possible, we included data for the vertigo outcomes that encompassed all of these three aspects (frequency, duration and severity/intensity of symptoms). However, we anticipated that these data may not be available from all studies. We therefore extracted data on the frequency of vertigo episodes as an alternative measure for these outcomes. 

##### Secondary outcomes

- Disease‐specific health‐related quality of life
  - Measured with the Dizziness Handicap Inventory (DHI, Jacobsen 1990), a validated measurement scale in widespread use. If data from the DHI were unavailable we extracted data from alternative validated measurement scales, according to the order of preference described in the list below (based on the validity of the scales for this outcome):
    - DHI short form (Tesio 1999);
    - DHI screening tool (Jacobsen 1998);
    - Vertigo Handicap Questionnaire (Yardley 1992a);
    - Meniere's Disease Patient Oriented Symptoms Index (MDPOSI, Murphy 1999);
    - University of California Los Angeles Dizziness Questionnaire (UCLADQ, Honrubia 1996);
    - AAO‐HNS Functional Level Scale (FLS, AAO‐HNS 1995).
- Hearing
  - Measured with pure tone audiometry and reported as the change in pure tone average (PTA), or (alternatively) by patient report, if data from PTA were not available.
- Tinnitus
  - Measured using any validated, patient‐reported questionnaire relating to the impact of tinnitus, for example the Tinnitus Handicap Inventory (THI, Newman 1996) or the Tinnitus Functional Index (TFI, Meikle 2012).
- Other adverse effects
  - Measured as the number of participants who experience at least one episode of the specified adverse events during the follow‐up period. Including the number of participants with the following specified adverse effects:
    - tympanic membrane perforation;
    - ear pain;
    - post‐injection vertigo;
    - new onset, permanent and total hearing loss in the affected ear;
    - new onset of tinnitus in the affected ear.

### Search methods for identification of studies

The Cochrane ENT Information Specialist conducted systematic searches for randomised controlled trials and controlled clinical trials in October 2021 and September 2022. There were no language, publication year or publication status restrictions. The date of the search was 14 September 2022. 

#### Electronic searches

The Information Specialist searched:

- the Cochrane ENT Trials Register (search via the Cochrane Register of Studies to 14 September 2022);
- the Cochrane Central Register of Controlled Trials (CENTRAL) (search via the Cochrane Register of Studies to 14 September 2022);
- Ovid MEDLINE(R) Epub Ahead of Print, In‐Process & Other Non‐Indexed Citations, Ovid MEDLINE(R) Daily and Ovid MEDLINE(R) (1946 to 14 September 2022);
- Ovid Embase (1974 to 14 September 2022);
- Web of Knowledge, Web of Science (1945 to 14 September 2022);
- ClinicalTrials.gov, www.clinicaltrials.gov (to 14 September 2022);
- World Health Organization (WHO) International Clinical Trials Registry Platform (ICTRP), https://trialsearch.who.int/ (to 14 September 2022).

The Information Specialist modelled subject strategies for databases on the search strategy designed for CENTRAL. The strategy has been designed to identify all relevant studies for a suite of reviews on various interventions for Ménière's disease. Where appropriate, they were combined with subject strategy adaptations of the highly sensitive search strategy designed by Cochrane for identifying randomised controlled trials and controlled clinical trials (as described in the *Cochrane Handbook for Systematic Reviews of Interventions* Version 5.1.0, Box 6.4.b, Handbook 2011). Search strategies for major databases including CENTRAL are provided in Appendix 2.

#### Searching other resources

We scanned the reference lists of identified publications for additional trials and contacted trial authors where necessary. In addition, the Information Specialist searched Ovid MEDLINE to retrieve existing systematic reviews relevant to this systematic review, so that we could scan their reference lists for additional trials. In addition, the Information Specialist ran a non‐systematic search of Google Scholar to identify trials not published in mainstream journals. 

We did not perform a separate search for adverse effects. We considered adverse effects described in included studies only.

### Data collection and analysis

#### Selection of studies

The Cochrane ENT Information Specialist used the first two components of Cochrane's Screen4Me workflow to help assess the search results: 

1. Known assessments – a service that matches records in the search results to records that have already been screened in Cochrane Crowd and been labelled as 'a RCT' or as 'not a RCT'. 
2. The machine learning classifier (RCT model) (Wallace 2017), available in the Cochrane Register of Studies (CRS‐Web), which assigns a probability of being a true RCT (from 0 to 100) to each citation. Citations that were assigned a probability score below the cut‐point at a recall of 99% were assumed to be non‐RCTs. We manually dual screened the results for those that scored on or above the cut‐point. 

At least two review authors (AL, KW) or co‐workers (BG, KG, SC, listed in Acknowledgements) independently screened the remaining titles and abstracts using Covidence, to identify studies that may be relevant for the review. Any discrepancies were resolved by consensus, or by retrieving the full text of the study for further assessment. 

We obtained the full text for any study that was considered possibly relevant and two authors (AL, KW) or co‐workers (BG, KG) again independently checked this to determine whether it met the inclusion criteria for the review. Any differences were resolved by discussion and consensus, or through recourse to a third author if necessary. 

We listed excluded any studies that were retrieved in full text but subsequently deemed to be inappropriate for the review (according to the inclusion/exclusion criteria), according to the main reason for exclusion. 

The unit of interest for the review is the study, therefore multiple papers or reports of a single study are grouped together under a single reference identification. The process for study selection is recorded in Figure 1. 

**1:**
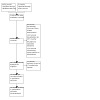


##### Screening eligible studies for trustworthiness

We assessed studies meeting our inclusion criteria for trustworthiness using a screening tool developed by Cochrane Pregnancy and Childbirth. This tool includes specified criteria to identify studies that are considered sufficiently trustworthy to be included in the review (see Appendix 3 and Figure 2). If studies were assessed as being potentially 'high‐risk', we attempted to contact the study authors to obtain further information or address any concerns. We planned to exclude studies from the main analyses of the review if there were persisting concerns over trustworthiness, or we were unable to contact the authors. However, over the course of the review it became apparent that the majority of included studies had some concerns ‐ typically due to missing information that was not reported in the original study publications. 

**2:**
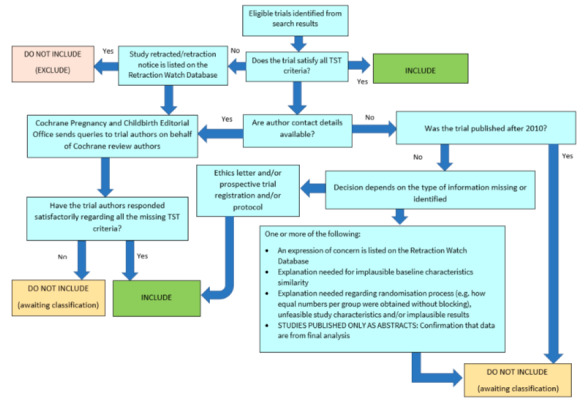
Cochrane Pregnancy and Childbirth Trustworthiness Screening Tool

Two included studies had no concerns when using the tool (Lambert 2016; NCT02265393). Three studies were published after 2010 but did not have a registered protocol, or the authors were unable to supply us with a copy of the trial protocol (Borghei 2016; El Shafei 2020; Ul Shamas 2017). Three studies had an equal number of participants allocated to each group, but did not report the use of blocked randomisation, which may highlight a concern with the randomisation process (El Shafei 2020; Garduno‐Anaya 2005; Ul Shamas 2017). Three studies provided very limited baseline information on participants with Ménière's disease, which was insufficient for us to determine whether there may have been issues with randomisation (AVERTS‐2; Borghei 2016; Ul Shamas 2017). Four studies reported no loss to follow‐up at all (Borghei 2016; El Shafei 2020; Lambert 2012; Ul Shamas 2017). 

We were unable to assess two studies, as no published results were available (AVERTS‐1; NCT03664674).

We attempted to contact study authors to clarify these issues, but we either received no reply, or the authors were unable to access the original trial data to clarify our queries. 

There are several possible explanations for the large number of studies that had concerns when using the tool. One is that there are issues with the trustworthiness of the studies identified in this review, and the data included may not give reliable estimates of the true effect. Alternatively, the trustworthiness screening tool may be excessively sensitive, and flag studies that are trustworthy, but where information has not been fully reported. We note that this tool (and others used for the same purpose) has not yet been validated for use. 

We therefore took the decision to include the studies in the review, despite the potential concerns over trustworthiness. The uncertainty in the results is captured as part of our GRADE rating of the certainty of the evidence, using the domain 'study limitations'. 

#### Data extraction and management

Two review authors (AL, KW) independently extracted outcome data from each study using a standardised data collection form. Where a study had more than one publication, we retrieved all publications to ensure complete extraction of data. Any discrepancies in the data extracted by the two authors were checked against the original reports, and differences were resolved through discussion and consensus. If required, we contacted the study authors for clarification.

We extracted data on the key characteristics of the studies, including the following information:

- study design, duration of the study, number of study centres and location, study setting and dates of the study;
- information on the participants, including the number randomised, those lost to follow‐up or withdrawn, the number analysed, the age of participants, gender, severity of the condition, diagnostic criteria used, inclusion and exclusion criteria for the individual studies;
- details of the intervention, comparator, and concomitant treatments or excluded medications;
- the outcomes specified and reported by the study authors, including the time points;
- funding for the study and any conflicts of interest for the study authors;
- information required to assess the risk of bias in the study, and to enable GRADE assessment of the evidence.

Once the extracted data were checked and any discrepancies resolved, a single author transferred the information to Review Manager 5 (RevMan 2020). 

The primary effect of interest for this review is the effect of treatment assignment (which reflects the outcomes of treatment for people who were assigned to the intervention) rather than a per protocol analysis (the outcomes of treatment only for those who completed the full course of treatment as planned). For the outcomes of interest in this review, we extracted the findings from the studies on an available case basis, i.e. all available data from all participants at each time point, based on the treatment to which they were randomised. This was irrespective of compliance, or whether participants had received the intervention as planned.

In addition to extracting pre‐specified information about study characteristics and aspects of methodology relevant to risk of bias, we extracted the following summary statistics for each study and outcome:

- For continuous data: the mean values, standard deviation and number of patients for each treatment group at the different time points for outcome measurement. Where change‐from‐baseline data were not available, we extracted the values for endpoint data instead. If values for the individual treatment groups were not reported, where possible we extracted summary statistics (e.g. mean difference) from the studies.
- For binary data: we extracted information on the number of participants experiencing an event, and the number of participants assessed at that time point. If values for the individual treatment groups were not reported, where possible we extracted summary statistics (e.g. risk ratio) from the studies.
- For ordinal scale data: if the data appeared to be normally distributed, or if the analysis performed by the investigators indicated that parametric tests are appropriate, then we treated the outcome measure as continuous data. Alternatively, if data were available, we converted these to binary data for analysis ‐ for example, for analysis of improvement in vertigo, when rated using the AAO‐HNS 1995 control of vertigo scale. 
- For time‐to‐event data: we did not identify any time‐to‐event data for the outcomes specified in the review. 

If necessary, we converted data found in the studies to a format appropriate for meta‐analysis, according to the methods described in the *Cochrane Handbook for Systematic Reviews of Interventions* (Handbook 2021). 

We pre‐specified time points of interest for the outcomes in this review. Where studies reported data at multiple time points, we took the longest available follow‐up point within each of the specific time frames. For example, if a study reported an outcome at 12 weeks and 20 weeks of follow‐up then the 20‐week data was included for the time point 3 to 6 months (12 to 24 weeks).

#### Assessment of risk of bias in included studies

Two authors (AL, KW) undertook assessment of the risk of bias of the included studies independently, with the following taken into consideration, as guided by the *Cochrane Handbook for Systematic Reviews of Interventions* (Handbook 2011):

- sequence generation;
- allocation concealment;
- blinding;
- incomplete outcome data;
- selective outcome reporting; and
- other sources of bias.

We used the Cochrane risk of bias tool (Handbook 2011), which involves describing each of these domains as reported in the study and then assigning a judgement about the adequacy of each entry: 'low', 'high' or 'unclear' risk of bias.

#### Measures of treatment effect

We summarised the effects of the majority of dichotomous outcomes (e.g. serious adverse effects) as risk ratios (RR) with 95% confidence intervals (CIs). We have also expressed the results as absolute numbers based on the pooled results and compared to the assumed risk in the summary of findings table (Table 1) and full GRADE profile (Table 2).

**1:** GRADE profile: intratympanic corticosteroids for Ménière's disease

**Certainty assessment**							**Number of participants**		**Effect**		**Certainty**	**Comment**
**№ of studies**	**Study design**	**Risk of bias**	**Inconsistency**	**Indirectness**	**Imprecision**	**Other considerations**	**Intratympanic corticosteroids**	**Placebo/no treatment**	**Relative** **(95% CI)**	**Absolute** **(95% CI)**		
**Improvement in vertigo frequency (follow‐up: range 6 months to ≤ 12 months; assessed with: AAO‐HNS 1995 Class A, B or C)**												
2	Randomised trials	Serious^a^	Not serious	Not serious	Serious^b^	None	30/31 (96.8%)	28/29 (96.6%)	**RR 1.00** (0.92 to 1.10)	**0 fewer per 1000** (from 77 fewer to 97 more)	⨁⨁◯◯ Low	
**Improvement in vertigo frequency (follow‐up: range ≥ 12 months; assessed with: AAO‐HNS 1995 Class A, B or C)**												
2	Randomised trials	Serious^a^	Not serious	Not serious	Serious^b^	None	31/31 (100.0%)	26/27 (96.3%)	**RR 1.03** (0.87 to 1.23)	**29 more per 1000** (from 125 fewer to 221 more)	⨁⨁◯◯ Low	
**Improvement in vertigo frequency: sensitivity analysis for complete/substantial improvement (follow‐up: range 3 months to < 6 months; assessed with: AAO‐HNS 1972 criteria: complete resolution of vertigo)**												
1	Randomised trials	Very serious^c^	Not serious	Serious^d^	Serious^b^	None	15/18 (83.3%)	13/18 (72.2%)	**RR 1.15** (0.81 to 1.64)	**108 more per 1000** (from 137 fewer to 462 more)	⨁◯◯◯ Very low	
**Improvement in vertigo frequency: sensitivity analysis for complete/substantial improvement (follow‐up: range 6 months to ≤ 12 months; assessed with: AAO‐HNS Class A or B, or complete resolution)**												
3	Randomised trials	Serious^e^	Not serious	Not serious	Serious^b^	None	43/49 (87.8%)	34/47 (72.3%)	**RR 1.23** (1.01 to 1.48)	**166 more per 1000** (from 7 more to 347 more)	⨁⨁◯◯ Low	
**Improvement in vertigo frequency: sensitivity analysis for complete/substantial improvement (follow‐up: range > 12 months; assessed with: AAO‐HNS 1995 Class A or B)**												
2	Randomised trials	Serious^e^	Not serious	Not serious	Serious^b^	None	31/31 (100.0%)	20/27 (74.1%)	**RR 1.30** (1.02 to 1.65)	**222 more per 1000** (from 15 more to 481 more)	⨁⨁◯◯ Low	
**Change in vertigo (global score) (follow‐up: range 3 months to <6 months; assessed with: change from baseline in 'Gates Score'; scale from: 0 to 4)**												
1	Randomised trials	Not serious	Not serious	Serious^f^	Serious^b^	Publication bias strongly suspected^g^	30	14	—	MD **0.13 points lower** (0.42 lower to 0.16 higher)	⨁◯◯◯ Very low	
**Change in vertigo (frequency) (follow‐up: range 3 months to < 6 months; assessed with: change from baseline in proportion of days with definitive vertigo episodes)**												
3	Randomised trials	Not serious	Not serious	Not serious	Serious^b^	Publication bias strongly suspected^g^	193	179	—	MD **0.05 points (proportion of days affected by vertigo) lower** (0.07 lower to 0.02 lower)	⨁⨁◯◯ Low	
**Change in vertigo (frequency) (follow‐up: range 6 months to ≤ 12 months; assessed with: change in number of episodes per month)**												
1	Randomised trials	Serious^h^	Not serious	Not serious	Very serious^i^	None	11	9	—	MD **0.1 episodes per month lower** (0.79 lower to 0.59 higher)	⨁◯◯◯ Very low	
**Change in vertigo (frequency) (follow‐up: range > 12 months; assessed with: change in number of episodes per month)**												
1	Randomised trials	Serious^h^	Not serious	Not serious	Very serious^i^	None	11	7	—	MD **0.07 episodes per month lower** (0.84 lower to 0.7 higher)	⨁◯◯◯ Very low	
**Serious adverse events**												
4	Randomised trials	Not serious	Not serious	Not serious	Very serious^b,j^	Publication bias strongly suspected^g^	9/296 (3.0%)	9/204 (4.4%)	**RR 0.64** (0.22 to 1.85)	**16 fewer per 1000** (from 34 fewer to 38 more)	⨁◯◯◯ Very low	
**Disease‐specific health‐related quality of life (follow‐up: range 3 months to < 6 months; assessed with: MDPOSI; scale from: 0 to 80, higher score = worse)**												
1	Randomised trials	Not serious	Not serious	Not serious	Very serious^i,k^	Publication bias strongly suspected^g^	14	30	—	Quote: "No changes in quality of life as measured by the MDPOSI total score were observed."	⨁◯◯◯ Very low	
**Disease‐specific health‐related quality of life (follow‐up: range 6 months to ≤ 12 months; assessed with: AAO‐HNS Functional Level Scale; scale from: 1 to 6, higher score = worse)**												
1	Randomised trials	Serious^h^	Not serious	Not serious	Very serious^i,j^	None	11	9	—	MD **0.38 points lower** (1.56 lower to 0.8 higher)	⨁◯◯◯ Very low	
**Disease‐specific health‐related quality of life (follow‐up: range > 12 months; assessed with: AAO‐HNS Function Level Scale; Scale from: 1 to 6, higher score = worse)**												
1	Randomised trials	Serious^h^	Not serious	Not serious	Very serious^i,j^	None	11	7	—	MD **0.45 points lower** (2.03 lower to 1.13 higher)	⨁◯◯◯ Very low	
**Change in hearing (follow‐up: range 6 months to ≤ 12 months; assessed with: hearing threshold (dB) with pure tone audiogram)**												
1	Randomised trials	Serious^h^	Not serious	Not serious	Very serious^i^	None	11	9	—	MD **4.95 dB lower** (16.5 lower to 6.6 higher)	⨁◯◯◯ Very low	
**Change in hearing (follow‐up: range > 12 months; assessed with: hearing threshold (dB) with pure tone audiogram)**												
1	Randomised trials	Serious^h^	Not serious	Not serious	Very serious^i^	None	11	7	—	MD **2.84 dB lower** (9.61 lower to 3.93 higher)	⨁◯◯◯ Very low	
**Change in hearing: improvement in hearing (follow‐up: range 3 months to < 6 months; assessed with: change in PTA of > 10 dB)**												
2	Randomised trials	Serious^l^	Not serious	Not serious	Serious^b^	Publication bias strongly suspected^g^	6/93 (6.5%)	13/91 (14.3%)	**RR 0.45** (0.18 to 1.15)	**79 fewer per 1000** (from 117 fewer to 21 more)	⨁◯◯◯ Very low	
**Change in hearing: improvement in hearing (follow‐up: range 6 months to ≤ 12 months; assessed with: change in PTA of > 10 dB)**												
2	Randomised trials	Serious^m^	Not serious	Not serious	Very serious^b,j^	None	14/38 (36.8%)	19/38 (50.0%)	**RR 0.73** (0.45 to 1.17)	**135 fewer per 1000** (from 275 fewer to 85 more)	⨁◯◯◯ Very low	
**Change in hearing: improvement in hearing (follow‐up: range > 12 months; assessed with: change in PTA of > 10 dB)**												
2	Randomised trials	Serious^a^	Not serious	Not serious	Very serious^b,j^	None	11/31 (35.5%)	13/27 (48.1%)	**RR 0.79** (0.46 to 1.35)	**101 fewer per 1000** (from 260 fewer to 169 more)	⨁◯◯◯ Very low	
**Change in tinnitus (follow‐up: range 3 months to < 6 months; assessed with: THI; scale from: 0 to 100, higher scores = worse)**												
1	Randomised trials	Not serious	Not serious	Not serious	Serious^b^	publication bias strongly suspected^g^	30	14	—	MD **9.69 points lower** (20.28 lower to 0.89 higher)	⨁⨁◯◯ Low	
**Tinnitus (follow‐up: range 6 months to ≤ 12 months; assessed with: THI; scale from: 0 to 100, higher scores = worse)**												
1	Randomised trials	Serious^h^	Not serious	Not serious	Very serious^i,j^	None	11	9	—	MD **1.12 points lower** (24.75 lower to 22.51 higher)	⨁◯◯◯ Very low	
**Tinnitus (follow‐up: range > 12 months to 0; assessed with: THI; scale from: 0 to 100, higher scores = worse)**												
1	Randomised trials	Serious^h^	Not serious	Not serious	Very serious^i,j^	None	11	7	—	MD **6.6 points higher** (7.79 lower to 20.99 higher)	⨁◯◯◯ Very low	
**Other adverse effects ‐ persistent tympanic membrane perforation**												
3	Randomised trials	Not serious	Not serious	Not serious	serious^b^	Publication bias strongly suspected^g^	12/203 (5.9%)	0/117 (0.0%)	**Peto OR 5.71** (1.56 to 20.96)	Not estimable	⨁⨁◯◯ Low	
								1.0%		**45 more per 1000** (from 6 more to 165 more) with an assumed risk of 1% in the control group.		
**Other adverse effects ‐ ear pain**												
4	Randomised trials	Not serious	Not serious	Not serious	Very serious^b,j^	Publication bias strongly suspected^g^	15/295 (5.1%)	7/205 (3.4%)	**Peto OR 1.19** (0.47 to 3.04)	**6 more per 1000** (from 18 fewer to 63 more)	⨁◯◯◯ Very low	
**Other adverse effects ‐ post‐injection vertigo**												
3	Randomised trials	Not serious	Not serious	Not serious	Very serious^b,j^	Publication bias strongly suspected^g^	19/219 (8.7%)	4/127 (3.1%)	**Peto OR 1.78** (0.65 to 4.87)	**23 more per 1000** (from 11 fewer to 105 more)	⨁◯◯◯ Very low	
**Other adverse effects ‐ total hearing loss**												
2	Randomised trials	Not serious	Not serious	Not serious	Very serious^b,j^	Publication bias strongly suspected^g^	1/133 (0.8%)	0/39 (0.0%)	**Peto OR 3.47** (0.02 to 486.20)	**0 fewer per 1000** (from 0 fewer to 0 fewer)	⨁◯◯◯ Very low	
								1.0%		**24 more per 1000** (from 10 fewer to 821 more)		
**Other adverse effects ‐ new onset of tinnitus in the affected ear**												
2	Randomised trials	Not serious	Serious^n^	Not serious	Very serious^b,j^	Publication bias strongly suspected^g^	8/189 (4.2%)	4/113 (3.5%)	**Peto OR 1.09** (0.30 to 3.91)	**3 more per 1000** (from 25 fewer to 90 more)	⨁◯◯◯ Very low	

**AAO‐HNS:** American Academy of Otolaryngology ‐ Head and Neck Surgery; **CI:** confidence interval; **MD:** mean difference; **MDPOSI**: Ménière’s Disease Patient‐Oriented Symptom‐Severity Index; **OR:** odds ratio; **PTA:** pure tone audiometry; **RR:** risk ratio; **THI:** Tinnitus Handicap Inventory ^a^High risk of performance and detection bias in one study, and attrition bias the other study. ^b^Sample size fails to meet optimal information size (taken as < 400 participants for continuous outcomes, < 300 events for dichotomous outcomes). ^c^Risk of performance bias and other bias. ^d^Concern over population included ‐ limited information on diagnosis of Ménière's disease. Outcome is complete resolution of vertigo, not improvement. ^e^High risk of performance and detection bias in the study with the largest weight in the meta‐analysis. ^f^An unvalidated rating score was used to assess this outcome. ^g^We are aware of two unpublished trials from the same pharmaceutical company that apparently showed negative efficacy results. ^h^Risk of attrition bias. ^i^Extremely small sample size. ^j^Confidence interval ranges from potential benefit to potential harm. ^k^Narrative description only, unable to provide an estimate of the effect. ^l^Concerns of performance bias and other bias in one trial, selective reporting bias in the other. ^m^Risk of performance bias in both studies, other bias in one study and detection bias in one study. ^n^TheI^2^ value is 32%; direction of effect varies between the trials.

The reported event rate was zero for some outcomes. We therefore used the Peto odds ratio (OR) to analyse these data, according to the guidance in Xu 2021, as this should produce less biased estimates of the effect size when events are rare (as described in the Handbook 2021). 

For continuous outcomes, we expressed treatment effects as a mean difference (MD) with standard deviation (SD). We did not need to use standardised mean difference to pool any data. 

#### Unit of analysis issues

Ménière's disease is unlikely to be a stable condition, and interventions may not have a temporary effect. If cross‐over trials were identified then we planned to use only the data from the first phase of the study. If cluster‐randomised trials were identified then we would have ensured that analysis methods were used to account for clustering in the data (Handbook 2021). However, no cross‐over or cluster‐randomised trials were identified for inclusion.  

We identified two studies with three arms (Lambert 2012; Ul Shamas 2017). The two arms in Lambert 2012 related to the same comparison (3 mg dexamethasone and 12 mg dexamethasone), therefore we included these data by pooling the two intervention arms, to avoid double‐counting of any participants (according to methods in the Handbook 2021). Only two arms in Ul Shamas 2017 were relevant to this review (dexamethasone and placebo), therefore we disregarded the third arm, intratympanic gentamicin. These data are included in a companion review on intratympanic aminoglycosides for Ménière's disease (Webster 2021a).

#### Dealing with missing data

We planned to contact study authors via email whenever the outcome of interest was not reported, if the methods of the study suggest that the outcome had been measured. We did the same if not all data required for meta‐analysis were reported (for example, standard deviations), unless we were able to calculate them from other data reported by the study authors. 

We contacted the company responsible for the two unpublished studies (AVERTS‐1; NCT03664674), but they were unable to provide us with additional information on the results of these studies, or any further results from those studies that had been published (AVERTS‐2; Lambert 2012; Lambert 2016; NCT02265393). 

#### Assessment of heterogeneity

We assessed clinical heterogeneity by examining the included studies for potential differences between them in the types of participants recruited, interventions or controls used and the outcomes measured. This is highlighted in the Included studies section, below.

We used the I^2^ statistic to quantify inconsistency among the trials in each meta‐analysis. We also considered the P value from the Chi^2^ test. However, few meta‐analyses were conducted in the course of this review, and we did not identify any serious inconsistency. 

#### Assessment of reporting biases

We assessed reporting bias as within‐study outcome reporting bias and between‐study publication bias.

##### Outcome reporting bias (within‐study reporting bias)

We assessed within‐study reporting bias by comparing the outcomes reported in the published report against the study protocol or trial registry, whenever this could be obtained. If the protocol or trial registry entry was not available, we compared the outcomes reported to those listed in the methods section. If results are mentioned but not reported adequately in a way that allows analysis (e.g. the report only mentions whether the results were statistically significant or not), bias in a meta‐analysis is likely to occur. We then sought further information from the study authors. If no further information was found, we noted this as being a 'high' risk of bias with the risk of bias tool. If there was insufficient information to judge the risk of bias we noted this as an 'unclear' risk of bias (Handbook 2011). 

##### Publication bias (between‐study reporting bias)

We did not have sufficient studies to create funnel plots for any analysis. Two of the studies included in this review have not been fully published (AVERTS‐1; NCT03664674). The only information available is from a press release, which indicates that the trials showed negative efficacy results. We therefore have concerns that publication bias affects the results of this review ‐ these concerns are reflected in the GRADE assessment of the certainty of the evidence. 

#### Data synthesis

##### Meta‐analysis of numerical data

Where possible and appropriate (if participants, interventions, comparisons and outcomes were sufficiently similar in the trials identified) we conducted a quantitative synthesis of results. We conducted all meta‐analyses using RevMan 2020. We anticipated that the underlying effect of the intervention may vary between studies, due to differences between participants, settings and the interventions used for each study. We planned to use a random‐effects model for meta‐analysis and explore whether the use of a fixed‐effect model substantially alters the effect estimates (see Sensitivity analysis). However, we were only able to use the Peto odds ratio (OR) ‐ a fixed‐effect method ‐ for some meta‐analyses in this review, due to rare or zero events in at least one of the studies included in the analysis.

For dichotomous data, we analysed treatment differences as a risk ratio (RR) calculated using the Mantel‐Haenszel methods.

For continuous outcomes, if all data were from the same scale, we pooled mean follow‐up values with change‐from‐baseline data and reported this as a mean difference. We did not need to report standardised mean differences in this review.

Improvement in vertigo symptoms may be assessed using a variety of methods, which consider different aspects of vertigo. These include:

- frequency of vertigo episodes;
- duration of vertigo episodes;
- severity/intensity of vertigo episodes;
- a composite measure of all of these aspects:
  - for example, assessed with a global score ‐ such as "how troublesome are your vertigo symptoms?", rated on an ordinal scale.

For the outcomes "improvement in vertigo" and "change in vertigo", we prioritised outcome measures that use a composite score ‐ encompassing aspects of vertigo frequency, duration and severity/intensity. Examples of this may include a global rating scale of vertigo impact (rated from 0 to 10, where 0 is defined as no symptoms, and 10 is defined as the most troublesome symptoms) or the vertigo/balance subscale of the Vertigo Symptom Scale (Yardley 1992b), or Vertigo Symptom Scale Short Form (Yardley 1998). As data from composite scores were not available from the majority of studies, then we also included data on the frequency of vertigo episodes as an alternative measure.

##### Synthesis using other methods

If we were unable to pool numerical data in a meta‐analysis for one or more outcomes we planned to provide a synthesis of the results using alternative methods, following the guidance in chapter 12 of the Handbook 2021. However, this was not necessary, as results were typically provided by a single study. 

#### Subgroup analysis and investigation of heterogeneity

If statistical heterogeneity was identified for any comparisons, we planned to assess this considering the following subgroups:

- Different types of corticosteroid
- Different doses/frequency of administration
- Method of delivery
- Use of concomitant treatment
- Diagnosis of Ménière's disease

However, due to the paucity of data available, and the few meta‐analyses included in this review, we did not carry out any subgroup analysis. 

#### Sensitivity analysis

We planned to carry out a number of sensitivity analyses for the primary outcomes in this review. However, the paucity of data and the lack of meta‐analyses has meant that this was not possible. 

If few studies are identified for meta‐analysis, the random‐effects model may provide an inaccurate measure of the between‐studies variance. Therefore, we explored the impact of using a fixed‐effect model using a sensitivity analysis, and the results are very similar (Table 3). For some meta‐analyses we used the Peto OR (a fixed‐effect method) due to zero events in at least one of the study arms. For completeness, we compared these results to a random‐effects method using the Mantel‐Haenszel OR, but the results were also very similar (Table 3).

**2:** Sensitivity analyses

**Primary analysis**	**Sensitivity analysis result**	**Description of analysis**
Analysis 1.1 Improvement in vertigo frequency at 6 to ≤ 12 months	RR 1.01 (95% CI 0.90 to 1.13)	Fixed‐effect model
Analysis 1.1 Improvement in vertigo frequency at > 12 months	RR 1.05 (95% CI 0.83 to 1.68)	Fixed‐effect model
Analysis 1.4 Change in vertigo frequency at 3 to < 6 months	MD ‐0.05 (95% CI ‐0.07 to ‐0.02)	Fixed‐effect model
Analysis 1.6 Serious adverse events	RR 0.72 (95% CI 0.30 to 1.71)	Fixed‐effect model
Analysis 1.9 Improvement in hearing (dichotomous data) at 3 to < 6 months	RR 0.45 (95% CI 0.18 to 1.14)	Fixed‐effect model
Analysis 1.9 Improvement in hearing (dichotomous data) at 3 to < 6 months	RR 0.44 (95% CI 0.14 to 1.38)	Improvement in hearing using data from 1000 Hz for Lambert 2016. Random‐effects model.
Analysis 1.9 Improvement in hearing (dichotomous data) at 3 to < 6 months	RR 0.40 (95% CI 0.08 to 1.96)	Improvement in hearing using data from 2000 Hz for Lambert 2016. Random‐effects model.
Analysis 1.9 Improvement in hearing (dichotomous data) at 6 to ≤ 12 months	RR 0.74 (95% CI 0.45 to 1.21)	Fixed‐effect model
Analysis 1.9 Improvement in hearing (dichotomous data) at > 12 months	RR 0.82 (95% CI 0.48 to 1.42)	Fixed‐effect model
Analysis 1.11 Persistent tympanic membrane perforation	OR 4.38 (95% CI 0.77 to 24.98)	Mantel‐Haenszel OR with random‐effects model
Analysis 1.11 Ear pain	OR 0.92 (95% CI 0.27 to 3.09)	Mantel‐Haenszel OR with random‐effects model
Analysis 1.11 Post‐injection vertigo	OR 1.74 (95% CI 0.58 to 5.26)	Mantel‐Haenszel OR with random‐effects model
Analysis 1.11 New onset of tinnitus in the affected ear	OR 1.00 (95% CI 0.23 to 4.40)	Mantel‐Haenszel OR with random‐effects model

CI: confidence interval; MD: mean difference; OR: odds ratio; RR: risk ratio

If there was uncertainty over the diagnostic criteria used for participants in the studies (for example, if it was not clear whether participants were diagnosed using criteria that are analogous to the AAO‐HNS criteria) then we also planned to explore this by including/excluding those studies from the analysis. However, as noted above we had such sparse data in the review that we were unable to conduct these analyses. 

We used the Cochrane Pregnancy and Childbirth Screening Tool to identify any studies with concerns over the data available. We had intended that any studies identified by the tool would be excluded from the main analyses in the review, but that we would explore the impact of including the data from these studies through a sensitivity analysis. However, as noted above, we had some concerns over the use of this tool, and few studies were included in the review, therefore this sensitivity analysis was not conducted. 

We did conduct one sensitivity analysis that was not pre‐specified in our protocol (Webster 2021c). When drafting the protocol for this review we stated "improvement in vertigo" as our outcome. However, over the course of the review it became apparent that "any improvement" may not represent a meaningful improvement for people with Ménière's disease. For example, an individual who suffered 100 vertigo attacks per year at baseline and then only 99 attacks per year at follow‐up could be stated to have 'improved' ‐ although it is not clear whether the difference would be of any importance. 

For our main analysis for this outcome we considered 'any improvement' in vertigo, but we also conducted a sensitivity analysis to see if the effect estimates were altered if we considered 'substantial improvement' in vertigo. 

#### Summary of findings and assessment of the certainty of the evidence

Two independent authors (AL, KW) used the GRADE approach to rate the overall certainty of evidence using GRADEpro GDT (https://gradepro.org/) and the guidance in chapter 14 of the *Cochrane Handbook for Systematic Reviews of Interventions* (Handbook 2021). Disagreements were resolved through discussion and consensus. The certainty of evidence reflects the extent to which we are confident that an estimate of effect is correct, and we have applied this in the interpretation of results. There are four possible ratings: high, moderate, low and very low. A rating of high certainty of evidence implies that we are confident in our estimate of effect and that further research is very unlikely to change our confidence in the estimate of effect. A rating of very low certainty implies that any estimate of effect obtained is very uncertain.

The GRADE approach rates evidence from RCTs that do not have serious limitations as high certainty. However, several factors can lead to the downgrading of the evidence to moderate, low or very low. The degree of downgrading is determined by the seriousness of these factors:

- Study limitations (risk of bias)
  - This was assessed using the rating from the Cochrane risk of bias tool for the study or studies included in the analysis. We rated down either one or two levels, depending on the number of domains that had been rated at high or unclear risk of bias. 
- Inconsistency
  - This was assessed using the I^2^ statistic and the P value for heterogeneity for all meta‐analyses, as well as by visual inspection of the forest plot. For results based on a single study we rated this domain as no serious inconsistency.
- Indirectness of evidence
  - We took into account whether there were concerns over the population included in these study or studies for each outcome, as well as whether additional treatments were offered that may impact on the efficacy of the intervention under consideration. 
- Imprecision
  - We took into account the sample size and the width of the confidence interval for each outcome. If the sample size did not meet the optimal information size (i.e. < 400 people for continuous outcomes or < 300 events for dichotomous outcomes), or the confidence interval crossed the small effect threshold we rated down one level. If the sample size did not meet the optimal information size and the confidence interval included both potential harm and potential benefit we rated down twice. We also rated down twice for very tiny studies (e.g. 10 to 15 participants in each arm), regardless of the estimated confidence interval.
- Publication bias
  - We considered whether there were likely to be unpublished studies that may impact on our confidence in the results obtained. 

We used a minimally contextualised approach and rated the certainty in the interventions having an important effect (Zeng 2021). Where possible, we used agreed minimally important differences (MIDs) for continuous outcomes as the threshold for an important difference. Where no MID was identified, we provide an assumed MID based on agreement between the authors. For dichotomous outcomes, we looked at the absolute effects when rating imprecision, but also took into consideration the GRADE default approach (rating down when a RR crosses 1.25 or 0.80). We have justified all decisions to downgrade the certainty of the evidence using footnotes, and added comments to aid the interpretation of the findings, where necessary. 

We provide a summary of findings tables for the only comparison:

- Intratympanic corticosteroids versus placebo/no treatment

We have included all primary outcomes in the summary of findings table. We planned to prioritise outcomes at the time point three to six months for presentation in the tables. However, no data were available at these time points for some outcomes, therefore we have shown the data for longer periods of follow‐up. We have also included a full GRADE profile for all results (see Table 2).

## Results

### Description of studies

#### Results of the search

The searches in September 2022 retrieved a total of 4434 records. This reduced to 3408 after the removal of duplicates. The Cochrane ENT Information Specialist sent all 3408 records to the Screen4Me workflow. The Screen4Me workflow identified 122 records as having previously been assessed: 83 had been rejected as not RCTs and 39 had been assessed as possible RCTs. The RCT classifier rejected an additional 1427 records as not RCTs (with 99% sensitivity). We did not send any records to the Cochrane Crowd for assessment. Following this process, the Screen4Me workflow had rejected 1510  records and identified 1898 possible RCTs for title and abstract screening. 

**Table:** 

** **	**Possible RCTs**	**Rejected**
Known assessments	39	83
RCT classifier	1859	1427
Total	1898	1510

We identified 89 additional duplicates. We screened the titles and abstracts of the remaining 1809 records. We discarded 1773 records and assessed 36 full‐text records.

We excluded 17 records (linked to 17 studies) with reasons recorded in the review (see Excluded studies). 

We included 10 completed studies (19 records) where some results were available. We also identified two additional records relating to two of these studies. 

A flow chart of study retrieval and selection is provided in Figure 1.

#### Included studies

We included a total of 10 RCTs (AVERTS‐1; AVERTS‐2; Borghei 2016; El Shafei 2020; Garduno‐Anaya 2005; Lambert 2012; Lambert 2016; NCT02265393; NCT03664674; Ul Shamas 2017). Details of individual studies can be found in the Characteristics of included studies table. 

##### Study design

All included studies were described as randomised controlled trials. Most were two‐arm trials, comparing an active intervention to either placebo or no treatment. Two studies were three‐arm trials (El Shafei 2020; Ul Shamas 2017). The study Ul Shamas 2017 included one group of participants who received intratympanic gentamicin. This intervention is not relevant for this review, but is assessed as part of a companion review on intratympanic aminoglycosides for Ménière's disease (Webster 2021a). The study El Shafei 2020 included two different methods of administering intratympanic corticosteroids ‐ either as drops through a ventilation tube, or as an intratympanic injection. However, there was no appropriate placebo arm for those who received intratympanic injections of corticosteroids, therefore for the purposes of this review we have included the data that compares intratympanic corticosteroid drops to intratympanic placebo drops. 

The duration of follow‐up for the studies ranged from a minimum of 12 weeks (3 months, AVERTS‐1; AVERTS‐2; Lambert 2012; NCT03664674) to a maximum of 24 months (Garduno‐Anaya 2005). The largest trial was AVERTS‐2, which randomised 174 participants, and the smallest was Garduno‐Anaya 2005, which randomised 22 participants. 

##### Participants

All the included studies recruited adult participants with a diagnosis of Ménière's disease. 

###### Diagnosis of Ménière's disease

For most studies, the diagnosis was made according to the AAO‐HNS 1995 criteria. A single study did not state the use of these criteria for making a diagnosis of Ménière's disease (Borghei 2016), and instead simply stated that "all patients with intractable Ménière's disease" were included in the study. As the study was conducted in an ENT department, we assume that participants were appropriately investigated to confirm the diagnosis, but we have reflected this uncertainty in our GRADE assessment of the evidence (where relevant). 

Three studies explicitly stated that only participants with definite Ménière's disease were included (Garduno‐Anaya 2005; Lambert 2012; Lambert 2016). Two further studies are likely to have included participants with definite disease, as they stated that the AAO‐HNS 1995 criteria were used, and also required the presence of "documented asymmetric sensorineural hearing loss" (AVERTS‐1; AVERTS‐2; NCT03664674). The remaining trials did not comment on whether participants with probable disease were also included. 

###### Features of Ménière's disease

Most studies specifically stated that participants with only unilateral disease were included (AVERTS‐1; AVERTS‐2; Garduno‐Anaya 2005; Lambert 2012; Lambert 2016; NCT02265393; NCT03664674). The remaining studies did not state whether participants had unilateral or bilateral disease. 

The majority of studies gave no information regarding the duration of Ménière's symptoms. Where the duration of symptoms was reported, the majority of participants had experienced symptoms for up to five years. Most participants in Borghei 2016 had symptoms for five years or less (88.9%), with the majority being within two years of diagnosis (about 50%). This was similar for Lambert 2016, where around 60% of participants had symptoms for five years or less, but some had symptoms for over 15 years. Approximately half of the participants in El Shafei 2020 had symptoms for a year or less, and the remainder had been diagnosed within the past five years. 

Many studies indicated that participants must have failed some form of conservative or medical treatment before entering the trial. Participants in Borghei 2016 were given maintenance treatment of a low salt diet, betahistine, triamterene H, plus "anti‐vertigo and anti‐emetics" as needed. Those in Garduno‐Anaya 2005 must have had insufficient relief from caffeine and salt restrictions, plus a vasodilator and diuretic taken for six months before entry to the trial. Participants in Lambert 2012 must also have tried a low salt diet and/or diuretics for at least one month without relief. Two other studies simply stated that participants must have persistent symptoms despite medical management, but it was not clear what this entailed (El Shafei 2020; Ul Shamas 2017). Three further trials did not describe the treatment that participants were taking before entry to the trial but did state that they must be willing to maintain their current therapy throughout the duration of the trial (AVERTS‐2; Lambert 2016; NCT02265393). No information was available for AVERTS‐1 or NCT03664674.

Several studies indicated that participants must be experiencing active vertigo attacks when entering the trial. The frequency of these attacks varied across the studies, with a mean of one attack per month at baseline in Garduno‐Anaya 2005 and seven or eight episodes per month in Lambert 2012 and Lambert 2016. The frequency of vertigo at baseline was not described by the remaining studies. 

##### Interventions and comparisons

###### Intratympanic corticosteroids compared to no treatment/placebo

All the included studies considered a comparison of some form of intratympanic dexamethasone to placebo. However, the method of administration and dose provided varied greatly across the studies. 

Two studies administered dexamethasone through a ventilation tube, either as self‐administered drops, given on alternate days for three months (Borghei 2016), or as an injection, given once a week for three weeks (El Shafei 2020). The total dose of dexamethasone was not reported for these studies. 

The remaining studies all used an intratympanic injection, given directly through the tympanic membrane. One study used a 2 mg dose of dexamethasone (0.5 mL of a 4 mg/mL solution), given with a single injection (Ul Shamas 2017) and the other used approximately 2 mg dexamethasone (0.5 mL to 0.8 mL of a 4 mg/mL solution), but administered it daily for a total of five days (Garduno‐Anaya 2005). 

The six remaining studies were conducted by the same pharmaceutical company and considered the use of a specific drug known as OTO‐104 (or Otividex). This is a suspension of dexamethasone in a polymer that forms a gel at body temperature ‐ in principle, enabling the drug to stay *in situ* for longer. The first of these studies included a dose‐finding approach, randomising participants to receive either 3 mg or 12 mg of active drug, administered in a single, 200 microlitre injection (Lambert 2012). For the purposes of this review we have pooled the data from these two, active treatment arms. The trials that followed this all used a dose of 12 mg (AVERTS‐1; AVERTS‐2; Lambert 2016; NCT02265393; NCT03664674). All of these studies used a single intratympanic injection, except for NCT02265393. This was conducted as a safety study and used two doses of the study drug, given at three‐monthly intervals. However, it should be noted that development of this gel‐based formulation was discontinued, and the product is not commercially available for use. 

##### Outcomes

###### 1. Improvement in vertigo

####### 1.1. Global score

No studies reported the improvement of vertigo using a global score that considered the frequency, duration and intensity of vertigo attacks. 

####### 1.2. Frequency

When drafting the protocol for this review we stated "improvement in vertigo" as our outcome. However, over the course of the review it became apparent that "any improvement" may not represent a meaningful improvement for people with Ménière's disease. For example, an individual who suffered 100 vertigo attacks per year at baseline and then only 99 attacks per year at follow‐up could be stated to have 'improved' although it is not clear whether the difference would be of any importance. 

For our main analysis we have considered any improvement in vertigo, but we have also conducted a sensitivity analysis to see if the effect estimates are altered if we consider substantial improvement in vertigo. 

Two studies that assessed improvement in vertigo frequency used the AAO‐HNS 1995 "control of vertigo" scale (El Shafei 2020; Garduno‐Anaya 2005). The number of vertigo attacks in the interval after treatment is divided by the number of vertigo spells prior to treatment and multiplied by 100. The resulting number indicates the extent of ‘control of vertigo’ or CoV. The AAO‐HNS further divides the control of vertigo into classes, where class A (CoV = 0) represents a complete control of vertigo, class B (CoV 1% to 40%) represents a substantial control of vertigo, class C (41% to 80%) limited control, class D (81% to 120%) insignificant control and class E (> 120%) worse control (deterioration).

One study used an adaptation of an earlier version of this scale, from the AAOO 1972 guidelines (Borghei 2016). This considers both vertigo and hearing loss. In brief, participants are assigned to Class A (absence of dizzy spells and improvement in hearing), Class B (absence of dizzy spells and no change in hearing), Class C (absence of dizzy spells and worsening of hearing) or Class D (failure to control dizzy episodes). An improvement in frequency of vertigo was considered to be any participant with Class A, B or C control. However, it should be noted that this actually represents a complete resolution of vertigo episodes, not simply a reduction in frequency. Borghei 2016 also included two additional categories: Class E (A or B criteria, but with recurrent vertigo) and Class F (C or D criteria, with recurrent vertigo). 

Improvement in vertigo frequency was not apparently assessed or reported by six studies (AVERTS‐1; AVERTS‐2; Lambert 2012; Lambert 2016; NCT02265393; Ul Shamas 2017).

###### 2. Change in vertigo

####### 2.1. Global score

A single study reported the change in vertigo using a global score that considered the frequency, duration and intensity of vertigo attacks (Lambert 2012). They used a score that was previously developed by Gates 2004 for a study of positive pressure treatment of Ménière's disease. Participants were asked to score vertigo‐free days as 0, days with a mild attack as 1, days with moderately severe attacks (lasting more than 20 minutes) as 2, days with severe attacks lasting longer than one hour (and accompanied by nausea and vomiting) as 3, and the worst attack ever experienced as 4. The total symptom score in a given period of time therefore incorporates aspects of the duration, frequency and severity of vertigo. We have been unable to establish whether this score is a validated method to measure vertigo severity and impact. 

No other studies considered the change in vertigo using a global score. 

####### 2.2. Frequency

Six studies considered the number of days with "definitive" episodes of vertigo, lasting > 20 minutes (AVERTS‐1; AVERTS‐2; Garduno‐Anaya 2005; Lambert 2012; Lambert 2016; NCT03664674). Two studies reported this as the proportion of days affected by vertigo (Lambert 2012; Lambert 2016). One study reported the number of days per month with definitive vertigo, but we have used these data to estimate the proportion of days affected, in order to pool the data from these trials (AVERTS‐2). One other study considered the number of definitive episodes of vertigo per month (Garduno‐Anaya 2005). Two studies only reported a statistical comparison between the groups, and did not fully report which measure was used to assess this (AVERTS‐1; NCT03664674).

Change in vertigo frequency was not reported by four studies (Borghei 2016; El Shafei 2020; NCT02265393; Ul Shamas 2017).

###### 3. Serious adverse events

Four studies considering OTO‐104 all fully reported serious adverse events (AVERTS‐2; Lambert 2012; Lambert 2016; NCT02265393). Two of the OTO‐104 studies stated that adverse effects would be assessed, but no data are reported (AVERTS‐1; NCT03664674). Three studies did not appear to systematically assess and report serious adverse events, but did provide a small amount of information (Borghei 2016; Garduno‐Anaya 2005; Ul Shamas 2017). However, this was reported in such a way that it could not be pooled with other data because it was not clear to which group participants experiencing the event were allocated (Borghei 2016; Ul Shamas 2017), or we could not be confident that data for serious adverse events were systematically collected (Garduno‐Anaya 2005). 

Finally, one study did not provide any information on serious adverse events, therefore we are uncertain whether no events occurred, or data on adverse events were not collected (El Shafei 2020). 

###### 4. Disease‐specific health‐related quality of life

This outcome was inconsistently assessed and reported across the included studies, and relevant numerical data were very sparse. Four studies did not apparently assess disease‐specific health‐related quality of life at all (Borghei 2016; El Shafei 2020; NCT02265393; Ul Shamas 2017). Lambert 2012 used the Meniere's Disease Patient Oriented Symptoms Index (MDPOSI) to assess quality of life, but only provided a narrative summary of the results. Lambert 2016 did not use a disease‐specific measure of quality of life, but instead used the SF‐36 (a global quality of life score) therefore we were unable to include these data. The trial registration for three studies indicated that disease‐specific quality of life would be assessed, but no results are reported (AVERTS‐1; AVERTS‐2; NCT03664674). 

The only study to report relevant numerical data was Garduno‐Anaya 2005. This study used both the Dizziness Handicap Inventory (DHI) and the Functional Level Scale (FLS) to assess quality of life. 

###### 5. Hearing

Four studies assessed hearing as a dichotomous outcome using pure tone audiometry, and considered a change of ≥ 10 dB to be an improvement in hearing (Borghei 2016; El Shafei 2020; Garduno‐Anaya 2005; Lambert 2016). One study also considered hearing as a continuous outcome (i.e. mean change and standard deviation, Garduno‐Anaya 2005). 

One study only provided a narrative summary of hearing results, with no numeric data (Lambert 2012). The study Ul Shamas 2017 also provided some data on hearing, but considered the entire group of trial participants, so we were unable to compare those receiving intratympanic corticosteroids with those receiving placebo.

Three studies did not report hearing outcomes, although this had been listed in the trial registration documents as an outcome of interest (AVERTS‐1; AVERTS‐2; NCT03664674). One further trial of OTO‐104 did not report hearing data at the six‐month time point (which would have allowed for a comparison of active intervention and placebo), but only at the 12‐month time point (when all participants had received the active treatment, NCT02265393). 

###### 6. Tinnitus

Most studies that assessed tinnitus did so using the Tinnitus Handicap Inventory (THI) (Garduno‐Anaya 2005; Lambert 2012; Lambert 2016; Ul Shamas 2017). However, the study Lambert 2016 only provided a narrative summary for this outcome, and the study Ul Shamas 2017 only provided information on the number of people who "improved", without information on what was classed as an improvement. Therefore, we were unable to use these data in any meta‐analysis. 

Two studies used unvalidated scales to measure tinnitus, and it was not clear whether these really considered the impact of tinnitus on quality of life, therefore the data have not been included in this review (Borghei 2016; NCT02265393). 

Four studies did not apparently assess this outcome (AVERTS‐1; AVERTS‐2; El Shafei 2020; NCT03664674). 

###### 7. Other adverse effects

Most studies provided some information on adverse effects, but the specific outcomes of interest in this review (tympanic membrane perforation, ear pain or vertigo at the time of the injection, hearing loss and new‐onset tinnitus) were not addressed by all the studies. Furthermore, some studies did not provide information on which group participants who suffered an adverse effect were allocated to, therefore we were unable to provide a comparison of the intervention and placebo (Borghei 2016; Garduno‐Anaya 2005; Ul Shamas 2017). The two studies of OTO‐104 that remain unpublished did not report any details on adverse effects (AVERTS‐1; NCT03664674).

#### Excluded studies

After assessing the full text, we excluded 17 articles from this review. The main reason for exclusion for each article is listed below. 

Five studies were not randomised controlled trials (Guo 2016; Maksoud 2015; NCT02768662; Sakata 1988; Thabet 2008).

Two studies were excluded due to use of an intervention that was not suitable for this review (Kitahara 2007; Kitahara 2008). Both of these articles reported on the use of steroids instilled in the ear at the time of a surgical intervention (endolymphatic sac drainage). 

One study was excluded because it had very short follow‐up (Silverstein 1998). This cross‐over trial did use an intratympanic injection of dexamethasone, but participants were only followed up for one month before 'crossing over' to the alternative intervention (placebo). To be eligible for this review we considered trials with a minimum of three months duration of follow‐up. 

Finally, we identified a number of review articles that did not provide any primary outcome data. This included four narrative reviews (Conde 1965; Godlowski 1965; Patel 2017; Richards 1971), and five systematic reviews or meta‐analyses (Alles 2006; Dimitriadis 2017; Hao 2022; Phillips 2011; Syed 2015). We checked the reference lists of the systematic reviews and meta‐analyses, to ensure that we had already identified any relevant trials. 

### Risk of bias in included studies

Two studies remain unpublished ‐ the only data available are reported in a press release on the company website. Therefore these have been rated at unclear risk of bias for all domains, as we were unable to assess the methods and conduct of the studies (AVERTS‐1; NCT03664674).

See Figure 3 for the risk of bias graph (our judgements about each risk of bias item presented as percentages across all included studies) and Figure 4 for the risk of bias summary (our judgements about each risk of bias item for each included study). All the studies included had some concerns regarding the risk of bias, with at least one domain being rated at unclear or high risk of bias. 

**3:**
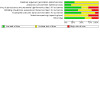
Risk of bias graph (our judgements about each risk of bias item presented as percentages across all included studies).

**4:**
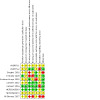
Risk of bias summary (our judgements about each risk of bias item for each included study).

#### Allocation

Three studies provided sufficient detail on the methods used for randomisation to confirm that an appropriate method was used (El Shafei 2020; Lambert 2016; NCT02265393). Two of these studies also provided detailed information on the methods used to ensure allocation was concealed, therefore we rated them at low risk of selection bias (Lambert 2016; NCT02265393). One study only provided information on generation of the random sequence, but did not describe methods used to conceal allocation, therefore we rated it at unclear risk of bias (El Shafei 2020).

Most of the included studies did not fully report the methods used for generation of a random sequence or methods used to conceal allocation, therefore we rated them at unclear risk of bias (AVERTS‐2; Borghei 2016; Garduno‐Anaya 2005; Lambert 2012; Ul Shamas 2017). It is possible that some of these studies did use an effective method to randomise participants, but as the methods were unclear or not described, the risk of bias has been rated as unclear. 

#### Blinding

The majority of studies explicitly stated that participants and study personnel were blinded to their treatment allocation, therefore we considered them to be at low risk of performance bias (AVERTS‐2; Garduno‐Anaya 2005; Lambert 2012; Lambert 2016; NCT02265393). One study stated that the participants were blinded to the intervention, but that study personnel were aware of group allocation, therefore we rated it as high risk (Borghei 2016). Two studies did not describe any attempts at blinding and, as the interventions were clearly different in the separate arms of the trial, we have assumed that participants and study personnel were aware of their treatment allocation, and rated this domain as high risk of bias (El Shafei 2020; Ul Shamas 2017).

Similarly, all four studies of OTO‐104 clearly indicated that those assessing outcomes (either study personnel or trial participants themselves) were blinded to treatment allocation, and we rated them at low risk of detection bias (AVERTS‐2; Lambert 2012; Lambert 2016; NCT02265393). We rated El Shafei 2020 and Ul Shamas 2017 at high risk of detection bias, as these studies were considered to be open‐label, with no blinding. We rated two studies at unclear risk of bias. In the study Borghei 2016, it was unclear who was responsible for assessing "improvement in vertigo" ‐ if this was self‐rated by participants then the outcome would be low risk, as participants were blinded to their group allocation. However, surgeons were aware of the treatment group, therefore there is the potential to introduce bias if they were responsible for assessing this outcome. Finally, in the study Garduno‐Anaya 2005, participants in the control arm were offered alternative treatments over the course of the trial, if their symptoms were not controlled. Therefore, it is possible that blinding of treatment allocation was not ensured over time, and that participants may have been aware of their group allocation at the time of outcome assessment.

#### Incomplete outcome data

We rated five studies at low risk of attrition bias, as either the majority of participants, or all participants, were included in the analysis (Borghei 2016; El Shafei 2020; Lambert 2012; Lambert 2016; NCT02265393). We rated two studies at unclear risk for this domain. No information on attrition was provided by Ul Shamas 2017. The AVERTS‐2 study was prematurely terminated by the study funder, due to negative efficacy results from a similar, unpublished trial. Therefore, a substantial number of participants enrolled in the study did not complete the full trial follow‐up. It is unclear whether they completed the 12‐week follow‐up period for the outcomes included in this review, or whether missing data were imputed as part of this analysis. 

Finally, we rated one study at high risk of attrition bias. The study Garduno‐Anaya 2005 discontinued follow‐up of participants in the control group if they received certain additional treatments. Therefore, by the 24‐month follow‐up period all 11 participants in the intervention group were available for follow‐up, but only 7 out of 11 in the control group provided data. We considered that the differential follow‐up presented a risk of bias in the results. 

#### Selective reporting

We rated all the included studies at either high or unclear risk of bias from selective reporting. Three studies did not have a published protocol to compare the reported results to, therefore we rated them at unclear risk of selective reporting (Borghei 2016; El Shafei 2020; Garduno‐Anaya 2005). We also rated two of the studies of OTO‐104 at unclear risk, due to issues in reporting: Lambert 2012 provided very limited, narrative data only on hearing and quality of life, precluding any meta‐analysis and NCT02265393 only reported most outcomes at 12 months of follow‐up (when all participants had received the study drug), rather than reporting at six‐month follow‐up (allowing a comparison of efficacy between control and intervention arms). 

Finally, we rated three studies at high risk of bias. The study Ul Shamas 2017 did not report any vertigo outcomes at all, which we considered to be extremely unusual for a trial of Ménière's disease. For AVERTS‐2 and Lambert 2016 there were discrepancies in reporting between the trial registration documents and the publications. The trial registry site for AVERTS‐2 stated that hearing and quality of life would be assessed, however these are not reported. Furthermore, tympanic membrane perforation is not included in the adverse event reporting, despite this being a widely recognised complication of this procedure. The trial registry site for Lambert 2016 indicated that outcomes would be reported after four months of follow‐up, but the trial results are all given at three months follow‐up. It is unclear whether this was a change in the analysis plan, or an error, but we rated it as a high risk of bias. 

#### Other potential sources of bias

No additional sources of bias were identified for the majority of included studies (AVERTS‐2; El Shafei 2020; Garduno‐Anaya 2005; Lambert 2012; Lambert 2016; NCT02265393). We had some concern over the outcome measures used by Borghei 2016, as well as concern over the definition of Ménière's disease, which was not clearly reported. We rated the study Ul Shamas 2017 at high risk of bias as very limited details were provided on the study methods, and data were not reported in a way that allowed adequate comparison of the intervention and control groups.

### Effects of interventions

See: Table 1

#### 1. Intratympanic steroids compared to no treatment/placebo

##### 1.1. Improvement in vertigo

For this outcome we included dichotomous data ‐ assessed as the proportion of participants whose vertigo had 'improved' or 'not improved'. 

###### 1.1.1. Improvement in global score

No studies measured the proportion of patients in whom there was an improvement in vertigo using a global score ‐ taking account of the frequency, severity or intensity and duration of symptoms. 

###### 1.1.2. Improvement in frequency

Two studies assessed improvement in the frequency of vertigo using the class of vertigo control, according to the AAO‐HNS 1995 criteria (El Shafei 2020; Garduno‐Anaya 2005). For this analysis we looked at the proportion of participants who had *any* improvement in the frequency of vertigo episodes (i.e. class A, B or C ‐ complete, substantial or limited control of vertigo). 

####### 1.1.2.1. At 3 to < 6 months

No data were reported at this time point. 

####### 1.1.2.2. At 6 to ≤ 12 months

Both studies reported data at 12 months. The risk ratio (RR) for improvement was 1.00 in those receiving intratympanic corticosteroids (95% confidence interval (CI) 0.92 to 1.10; 2 studies; 60 participants; I^2^ = 0%; low‐certainty evidence; Analysis 1.1).

####### 1.1.2.3. At >12 months

One study reported at 18 months (El Shafei 2020), and the other at 24 months (Garduno‐Anaya 2005). The risk ratio for improvement in those receiving intratympanic corticosteroids was 1.03 (95% CI 0.87 to 1.23; 2 studies; 58 participants; I^2^ = 30%; low‐certainty evidence; Analysis 1.1).

####### 1.1.2.4. Sensitivity analysis

Our protocol stated that this primary outcome measure should be any "improvement" in vertigo, therefore in the analyses above we have included data that considers participants who had *any* degree of improvement. However, we note that class C vertigo control includes a reduction in frequency of episodes of between 20% and 59%. We considered that a reduction of only 20% may not be viewed as an important change in the frequency of episodes by many people with Ménière's disease, or by healthcare professionals. Indeed, a number of publications considered only class A or B as 'improvement'. We also noted that the number of participants in the placebo groups who 'improved' was considerable. This makes it hard to discriminate between interventions. We therefore explored whether assessing those with complete or substantial control of vertigo would change our effect estimates. An additional study was included in these analyses (Borghei 2016). This study reported on improvement of vertigo using the AAOO 1972 criteria, therefore it only includes those with *complete* resolution of vertigo (not substantial). 

At 3 to < 6 months the RR was 1.15 for those receiving intratympanic corticosteroids (95% CI 0.81 to 1.64; 1 study; 36 participants; very low‐certainty evidence; Analysis 1.2). At 6 to ≤ 12 months the RR was 1.23 (95% CI 1.01 to 1.48; 3 studies; 96 participants; I^2^ = 0%; low‐certainty evidence;  Analysis 1.2). At > 12 months the RR was 1.30 (95% CI 1.02 to 1.65; 2 studies; 58 participants; I^2^ = 6%; low‐certainty evidence; Analysis 1.2). 

Although the certainty of the evidence is low throughout, this sensitivity analysis does indicate that the effect size may vary depending on how improvement is defined. There may be no difference in the chance of *any* improvement in the frequency of vertigo when using intratympanic corticosteroids. However, it may be that more people experience substantial or complete improvement with intratympanic corticosteroids than with placebo. 

##### 1.2. Change in vertigo

This outcome included data on the change in vertigo using a continuous numerical scale.

###### 1.2.1. Change in global score

A single study reported on the change in vertigo using a global score, which included the frequency of episodes, the severity or intensity of symptoms and the duration of episodes (Lambert 2012). Symptoms were reported using a scale originally developed and reported in Gates 2004. Symptoms were rated on a daily basis by study participants with a score of 0 to 4 (0 = vertigo‐free days; 1 = mild attack, 2 = moderately severe attack lasting more than 20 minutes, 3 = severe attack lasting an hour or more accompanied by nausea or vomiting, 4 = the worst attack ever experienced to date). We have been unable to identify whether this scoring system has been validated in any way. Lambert 2012 reported on the mean change from baseline in the vertigo score.

####### 1.2.1.1. At 3 to < 6 months

The mean difference in vertigo score was ‐0.13 for those receiving intratympanic corticosteroids (95% CI ‐0.42 to 0.16; 1 study; 44 participants; very low‐certainty evidence; Analysis 1.3). We considered that this was likely to represent a trivial difference between those receiving intratympanic corticosteroids and placebo, however the evidence was very uncertain. 

####### 1.2.1.2. At 6 to ≤ 12 months

No data were reported at this time point. 

####### 1.2.1.3. At > 12 months

No data were reported at this time point. 

###### 1.2.2. Change in vertigo frequency

Four studies reported on the change in vertigo frequency, although different measurements were used to assess the frequency of vertigo episodes. Lambert 2012 and Lambert 2016 both assessed the change in the *proportion of days* over one month that were affected by vertigo (i.e. a proportion of 1 means that vertigo occurs on every day, a proportion of 0.1 means that vertigo occurs on 1 in 10 days). The AVERTS‐2 study reported on the number of "definitive vertigo days" per month. To enable these data to be pooled, we converted the number of days into a proportion of days affected by vertigo in a one‐month period, assuming a 30‐day month. 

Garduno‐Anaya 2005 reported the actual number of vertigo episodes in one month. For this study, data on the number of vertigo episodes were reported for individual participants, therefore summary data for the change in number of episodes over time were calculated by members of the review team (KW, KG). 

The scale developed by Gates 2004 was used to assess the presence of vertigo (as described above) by Garduno‐Anaya 2005, Lambert 2012 and Lambert 2016. These studies counted "definitive vertigo episodes", as any day on which a score of at least 2 was recorded (a moderately severe attack lasting more than 20 minutes). The terminology "definitive vertigo episodes" was also used in the AVERTS‐2 study, and as this was conducted by the same company as Lambert 2012 and Lambert 2016 we have assumed that the same scoring system was used, although it is not explicit in the study data. 

####### 1.2.2.1. At 3 to < 6 months

Three studies reported data at three months (AVERTS‐2; Lambert 2012; Lambert 2016). The mean difference in the proportion of days per month affected by vertigo was ‐0.05 in those receiving intratympanic corticosteroids (95% CI ‐0.07 to ‐0.02; 3 studies; 372 participants; I^2^ = 0%; low‐certainty evidence; Analysis 1.4).  This would equate to a reduction in vertigo days of approximately 1.5 days per month with treatment (95% CI from a decrease of 0.6 days per month, to a decrease of 2.17 days per month). We considered that this would be a small, but potentially important change. 

However, the press release for the AVERTS‐1 study stated that, "The clinical trial missed its primary endpoint which was the count of definitive vertigo days by Poisson Regression analysis (p=0.62). Patients in both the OTIVIDEX and placebo groups showed similar reductions in the number and severity of vertigo episodes during the three month observation period. OTIVIDEX patients reported a 58% reduction from baseline in vertigo frequency in Month 3 vs. 55% for placebo patients". The change in vertigo frequency from baseline and average daily vertigo count were also not significantly different in those receiving OTO‐104, compared to those receiving placebo. 

Similarly, the press release for NCT03664674 states that "the Phase 3 clinical trial of OTIVIDEX in patients with Ménière’s disease did not achieve the primary endpoint, which was the count of definitive vertigo days (DVD) in Month 3 for OTIVIDEX vs. placebo for the intent‐to‐treat (ITT) population (n = 148; p value = 0.312) using the Negative Binomial Model".

The overall result for this outcome at this time point is clearly subject to publication bias ‐ two studies remain unpublished and their results cannot be incorporated into the meta‐analysis. We have attempted to reflect this with the GRADE certainty of the evidence, which has been lowered because of this bias. However, we would also advocate extreme caution in interpreting the results of this analysis.

####### 1.2.2.2. At 6 to ≤ 12 months

One study reported at this time point (Garduno‐Anaya 2005). The mean difference in the number of vertigo episodes per month at 12 months was ‐0.10 in those receiving intratympanic corticosteroids (95% CI ‐0.79 to 0.59; 1 study; 20 participants; very low‐certainty evidence; Analysis 1.5).

####### 1.2.2.3. At > 12 months

Garduno‐Anaya 2005 also reported at 24 months of follow‐up. The mean difference in the number of vertigo episodes per month at 24 months was ‐0.07 in those receiving intratympanic corticosteroids (95% CI ‐0.84 to 0.70; 1 study; 18 participants; very low‐certainty evidence; Analysis 1.5).

##### 1.3. Serious adverse events

Four studies systematically assessed and reported on the occurrence of serious adverse events, or treatment‐emergent adverse effects (AVERTS‐2; Lambert 2012; Lambert 2016; NCT02265393). However, no events were identified in one of these studies (Lambert 2012). The RR for serious adverse events in those receiving intratympanic corticosteroids was 0.64 (95% CI 0.22 to 1.85; 4 studies; 500 participants; I^2^ = 15%; very low‐certainty evidence; Analysis 1.6). 

Two further studies gave some narrative description of serious adverse events, but it is not clear whether serious adverse events were specifically assessed over the course of the studies. Borghei 2016 stated the following: "Complications: There was just one case of chronic infection with tympanic membrane perforation without resolution after treatment, which [...] underwent tympanoplasty surgery". However, it was unclear which group this participant was allocated to, and it should be noted that all participants in this trial had a ventilation tube inserted. Garduno‐Anaya 2005 stated, "We did not have any complications". Four studies did not provide any information on serious adverse events (AVERTS‐1; El Shafei 2020; NCT03664674; Ul Shamas 2017). 

##### 1.4. Disease‐specific health‐related quality of life

A single study provided numeric data for this analysis. Garduno‐Anaya 2005 assessed quality of life using the Functional Level Scale (FLS), described by the AAO‐HNS 1995. This is a six‐point scale that considers the impact of vertigo on quality of life and ability to engage in normal activities. A score of 1 indicates that dizziness has no effect on daily activities, a score of 6 indicates that the individual has been disabled for at least one year. It is not clear what would constitute an important difference on this scale, but we have assumed that a change of 1 point would be of importance to people with Ménière's disease.  

Lambert 2012 used the Meniere's Disease Patient Oriented Symptoms Index (MDPOSI) to assess disease‐specific health‐related quality of life, but only provided a narrative report of the outcome data. Lambert 2016 only assessed generic quality of life (using the SF‐36), and did not use a tool that specifically considered the impact of Ménière's disease on quality of life. The remaining studies did not report on disease‐specific quality of life (AVERTS‐1; AVERTS‐2; Borghei 2016; El Shafei 2020; NCT02265393; NCT03664674; Ul Shamas 2017).

###### 1.4.1. At 3 to < 6 months

No numeric data were reported at this time point. Lambert 2012 stated "No changes in quality of life as measured by the MDPOSI total score were observed. No differences were observed for any of the subscales of the [...] MDPOSI" (1 study; 44 participants; very low‐certainty evidence). 

###### 1.4.2. At 6 to ≤ 12 months

At 12 months, the mean difference in FLS score for those receiving intratympanic corticosteroids was ‐0.38 points (95% CI ‐1.56 to 0.80; 1 study; 20 participants; very low‐certainty evidence; Analysis 1.7). 

###### 1.4.3. At >12 months

The mean difference in FLS score at 24 months for those receiving intratympanic corticosteroids was ‐0.45 points (95% CI ‐2.03 to 1.13; 1 study; 18 participants; very low‐certainty evidence; Analysis 1.7). The same study reported a statistically significant difference in the DHI score at 24 months, favouring the intratympanic corticosteroid group, but the variance was not reported, and the difference between the two groups did not reach the minimally important difference for this measurement scale (mean score 8.3 in the intratympanic corticosteroid group, compared to 23.7 in the placebo group, MID = 18 points for the DHI) (Garduno‐Anaya 2005). 

##### 1.5. Change in hearing

The majority of studies reported some information regarding change in hearing, although the methods of reporting varied. 

Garduno‐Anaya 2005 provided individual participant data for the hearing threshold at baseline and at different follow‐up time points through the trial. From these data we were able to calculate the change in hearing threshold. 

Three studies did not provide the actual change in hearing threshold. Instead, participants were assessed with regard to hearing improvement. Borghei 2016 reported on the number of participants who had an improvement of > 10 dB in at least two different frequencies of a four‐frequency pure‐tone audiogram. El Shafei 2020 also used an improvement of > 10 dB, but looked at the pure‐tone average, assessed over four different frequencies. Lambert 2016 used a threshold of > 10 dB to identify "improvement" but reported this separately for the different frequencies assessed. For our analysis we considered an improvement in hearing at 500 Hz, but we conducted a sensitivity analysis to assess the impact of analysing data from different frequencies.

Lambert 2012 only provided a narrative summary of hearing outcomes. Some data on hearing was reported by Ul Shamas 2017, but results were given for the whole study population, therefore we were unable to compare outcomes for those who received and did not receive intratympanic corticosteroids. Similarly, data on hearing was reported in NCT02265393 at the 12‐month follow‐up point, meaning that we were unable to compare those who received intratympanic corticosteroids and placebo. All participants had received active treatment by this point.

Finally, the trial registration for AVERTS‐1, AVERTS‐2 and NCT03664674 indicated that hearing would be assessed with pure tone audiometry at three months, but no data are reported. 

###### 1.5.1. At 3 to < 6 months

No study reported on the change in hearing as a continuous outcome at this time point. 

Two studies did assess "improvement" in hearing at three to four months (Borghei 2016; Lambert 2016). The RR for improvement in those receiving intratympanic corticosteroids was 0.45 (95% CI 0.18 to 1.15; 2 studies; 184 participants; I^2^ = 0%; very low‐certainty evidence; Analysis 1.9). For this analysis we considered improvement in hearing at 500 Hz for Lambert 2016; however, a sensitivity analysis using data at 1000 Hz or 2000 Hz resulted in a widening of the confidence intervals, but no major differences in the overall result (Table 3). 

Lambert 2012 only provided a narrative description of hearing outcomes, stating: "There were no clinically meaningful changes observed in hearing at all frequencies, pure‐tone averages, or speech discrimination. There were no instances of persistent conductive hearing loss associated with OTO‐104 injection."

###### 1.5.2. At 6 to ≤ 12 months

One study reported on the mean change in hearing threshold at 12 months (Garduno‐Anaya 2005). The mean difference in hearing threshold for those receiving intratympanic corticosteroids was ‐4.95 dB (95% CI ‐16.50 to 6.60; 1 study; 20 participants; very low‐certainty evidence; Analysis 1.8).  

Two studies assessed "improvement" in hearing at 12 months (Borghei 2016; El Shafei 2020). The RR for improvement in those receiving intratympanic corticosteroids was 0.73 (95% CI 0.45 to 1.17; 2 studies; 76 participants; I^2^ = 0%; very low‐certainty evidence; Analysis 1.9). 

###### 1.5.3. At > 12 months

Garduno‐Anaya 2005 also reported on the mean change in hearing threshold at 24 months. The mean difference in hearing threshold for those receiving intratympanic corticosteroids was ‐2.84 (95% CI ‐9.61 to 3.93; 1 study; 18 participants; very low‐certainty evidence; Analysis 1.8). 

One study assessed "improvement" in hearing at 12 months (El Shafei 2020). The RR for improvement in those receiving intratympanic corticosteroids was 0.77 (95% CI 0.45 to 1.32; 1 study; 40 participants; very low‐certainty evidence; Analysis 1.9). 

##### 1.6. Change in tinnitus

Two studies assessed the impact of intratympanic corticosteroids on tinnitus (Garduno‐Anaya 2005; Lambert 2012). Both used the Tinnitus Handicap Inventory (THI), a questionnaire that considers the impact of tinnitus symptoms. The range of possible scores is from 0 to 100, and higher scores indicate more severe symptoms. A change of seven points has been suggested as a minimally important difference for this scale (Zeman 2011). 

###### 1.6.1. At 3 to < 6 months

The mean difference in the THI for those receiving intratympanic corticosteroids was a reduction of 9.69 points, which may suggest an important benefit with intratympanic corticosteroids (95% CI ‐20.28 to 0.89; 1 study; 44 participants; low‐certainty evidence; Analysis 1.10).

###### 1.6.2. At 6 to ≤ 12 months

The mean difference in the THI for those receiving intratympanic corticosteroids was ‐1.12 points (95% CI ‐24.75 to 22.51; 1 study; 20 participants; very low‐certainty evidence; Analysis 1.10). 

###### 1.6.3. At > 12 months

The mean difference in the THI for those receiving intratympanic corticosteroids was an increase of 6.60 points (95% CI ‐7.79 to 20.99; 1 study; 18 participants; very low‐certainty evidence; Analysis 1.10). 

##### 1.7. Other adverse events

Four studies fully reported some adverse events of interest to this review (AVERTS‐2; Lambert 2012; Lambert 2016; NCT02265393). One study stated "We did not have any complications", but it is not clear which adverse effects were specifically assessed and documented during the trial (Garduno‐Anaya 2005). One study did not report any information on adverse effects (El Shafei 2020). Two studies reported on the occurrence of some adverse effects, but did not state which group participants who experienced these adverse effects were allocated to, therefore we are unable to make a comparison of intratympanic corticosteroids and placebo (Borghei 2016; Ul Shamas 2017). Two studies did not report any information on adverse effects (AVERTS‐1; NCT03664674).

As most adverse effects were rare, and some studies reported zero events in at least one group, we have used the Peto odds ratio to analyse these data. We also note that these findings may not be easily generalisable to a non‐trial context. Many of these adverse effects are specifically related to intratympanic injection. In the context of these trials (where placebo injections are used as the comparator) it is therefore appropriate to provide a comparison of the groups. However, in clinical practice, the decision for people with Ménière's disease is whether to have an intratympanic injection or not. Therefore the absolute effects in the intervention group may be of more relevance when selecting a treatment.

###### 1.7.1. Persistent tympanic membrane perforation

The Peto odds ratio for persistent tympanic membrane perforation in those receiving intratympanic corticosteroids was 5.71 (95% CI 1.56 to 20.96; 3 studies; 320 participants; I^2^ = 0%; low certainty evidence; Analysis 1.11). This suggests that intratympanic corticosteroids may substantially increase the chance of a persistent tympanic membrane perforation when compared to placebo. The absolute effects in the included studies were: no perforations out of 117 participants in the control group (0%), compared to 12 perforations out of 203 participants in the intratympanic corticosteroids group (5.9%).

One further study described one instance of tympanic membrane perforation, but it is unclear which group this participant was allocated to, and all study participants in this trial had a ventilation tube inserted (Borghei 2016). 

###### 1.7.2. Ear pain

The Peto odds ratio for ear pain in those receiving intratympanic corticosteroids was 1.19 (95% CI 0.47 to 3.04; 4 studies; 500 participants; I^2^ = 0%; very low‐certainty evidence; Analysis 1.11). The absolute effects in the included studies were: 7 out of 205 participants in the control group (3.4%), compared to 15 out of 295 participants in the intratympanic corticosteroids group (5.1%).

###### 1.7.3. Post‐injection vertigo

Three studies reported on this outcome (AVERTS‐2; Lambert 2012; NCT02265393). The Peto odds ratio was 1.78 for those receiving intratympanic corticosteroids (95% CI 0.65 to 4.87; 1 study; 346 participants; very low‐certainty evidence; Analysis 1.11). The absolute effects in the included studies were: 4 out of 127 participants in the control group (3.1%), compared to 19 out of 219 participants in the intratympanic corticosteroids group (8.7%).

###### 1.7.4. New onset, permanent and total hearing loss in the affected ear

Two studies reported on total hearing loss following treatment (Lambert 2012; NCT02265393), although no events were reported by one study (Lambert 2012). The single occurrence of hearing loss reported by NCT02265393 was described as "unilateral deafness. Severe, definitely related", therefore we assume that this could be regarded as "new onset, permanent and total hearing loss in the affected ear". The Peto odds ratio was 3.47 (95% CI 0.02 to 486.20; 2 studies; 172 participants; very low‐certainty evidence; Analysis 1.11). The absolute effects in the included studies were: 0 out of 39 participants in the control group (0%), compared to 1 out of 133 participants in the intratympanic corticosteroids group (0.8%).

###### 1.7.5. New onset of tinnitus in the affected ear

Two studies also reported on this outcome (AVERTS‐2; NCT02265393). The NCT02265393 study only described this outcome as "tinnitus", but we have assumed that this represents new‐onset tinnitus in the affected ear. The Peto odds ratio was 1.09 (95% CI 0.30 to 3.91; 2 studies; 302 participants; I^2^ = 32%; very low‐certainty evidence; Analysis 1.11). The absolute effects in the included studies were: 4 out of 113 participants in the control group (3.5%), compared to 8 out of 189 participants in the intratympanic corticosteroids group (4.2%).

## Discussion

### Summary of main results

Intratympanic corticosteroids may make little or no difference to the proportion of people who experience any improvement in the frequency of vertigo at either 6 to ≤ 12 months, or ≥ 12 months. However, intratympanic corticosteroids may increase the number of people who experience either complete resolution of vertigo, or a substantial improvement in vertigo frequency, at 6 to ≤ 12 months or ≥ 12 months. The evidence for this was very uncertain at the earliest time period (3 to < 6 months).

When assessing vertigo by a change on a numerical scale, the evidence was very uncertain about the effect of intratympanic corticosteroids on a global score of vertigo severity. However, at 3 to < 6 months, intratympanic corticosteroids may slightly reduce the frequency of vertigo episodes, as compared to placebo. At later time points the evidence was very uncertain. 

The evidence on serious adverse effects was also very uncertain, so we are unsure whether these are affected by the use of intratympanic corticosteroids. We also found very low‐certainty evidence regarding disease‐specific health‐related quality of life, although any difference between the two groups appeared to be trivial. 

The data on hearing outcomes was rather mixed and still all very low‐certainty. Continuous data (reporting on hearing thresholds using pure tone average (PTA)) identified a trivial difference between those receiving intratympanic corticosteroids and those receiving placebo. However, for those studies that assessed hearing improvement (of > 10 dB on PTA), the proportion of people who improved was greater in those receiving placebo than in those receiving intratympanic corticosteroids at each time period, although the confidence intervals were wide (including the line of no effect, i.e. the result was not statistically significant), and the evidence was very low‐certainty.  

Intratympanic corticosteroids may slightly reduce the severity of tinnitus (as measured with the Tinnitus Handicap Inventory (THI)) at 3 to 6 months, but the data at later time points were very uncertain. 

Finally, intratympanic corticosteroids may result in an increase in the number of people who experience persistent tympanic membrane perforation. The evidence for other adverse effects (including ear pain, post‐injection vertigo, total hearing loss and new‐onset tinnitus) was very uncertain. 

### Overall completeness and applicability of evidence

We identified a number of studies assessing this intervention. However, we have significant concerns about the certainty of the findings from this review, because of unpublished data. Inclusion of these unpublished data may have altered our conclusions about the efficacy of intratympanic corticosteroids. Six studies included in this review were conducted by the same company (Otonomy), but two of these studies remain unpublished, and we have been unable to acquire the data to include in our review. 

It should be noted that these six studies all assess the same intervention, OTO‐104 ‐ a suspension of dexamethasone in a polymer that forms a gel at body temperature. As noted above, this formulation was discontinued by the company, and is not commercially available. It is possible that the efficacy and harms associated with this specific corticosteroid formulation differ from those seen with other preparations. Due to a paucity of data we were unable to conduct any subgroup analysis to determine whether this may be the case. 

In addition, participants in these six studies were followed up in their randomised groups for a maximum of four months. The only data regarding longer‐term outcomes for those using intratympanic corticosteroids come from smaller studies, many of which have a high risk of bias. There is more uncertainty in the results at later time periods. 

Despite an extensive search, we did not find any studies that considered other types of corticosteroid ‐ all included studies considered the use of dexamethasone. The dose of corticosteroid used varied from approximately 2 mg to a maximum of 12 mg. Therefore, the current evidence base relates only to dexamethasone when used at these doses. However, our protocol was designed to include any type of corticosteroid, used at any dose, therefore if relevant RCTs of different doses or of alternative corticosteroids existed these would have been included (Webster 2021c). 

Assessing adverse effects can be challenging. Many of the studies included in this review used intratympanic injections for administration of corticosteroids. This procedure may itself carry a risk of adverse effects ‐ such as ear discharge or tympanic membrane perforation ‐ regardless of the material injected. Therefore when balancing the risks and benefits of this procedure, individuals with Ménière's disease may wish to have information on the frequency with which these events occur as a consequence of intratympanic injection. In this review we identified low‐certainty evidence that intratympanic corticosteroids may increase the risk of persistent intratympanic membrane perforation. This may be expected in clinical practice, where the chance of spontaneous tympanic membrane perforation ‐ in the absence of any intervention ‐ is very rare. However, it should also be noted that participants in the control group of many of these studies also received a placebo injection. Therefore any increase in the risk of perforation seen here is potentially due to an additional risk from the injected material (i.e. corticosteroid), rather than from the procedure itself. 

It is noteworthy that ‐ in this situation ‐ evidence regarding the risks of an intervention may come from different types of studies to those which consider efficacy. Clearly, placebo interventions are required to appropriately consider the efficacy of an intervention such as intratympanic corticosteroids. However, when the procedure itself (intratympanic injection) is associated with specific risks, it is also relevant to compare the intervention to no treatment ‐ in order to appropriately gather information on the absolute risk of harms. 

This review was conducted as part of a suite considering different interventions for Ménière's disease. A number of issues were identified as affecting the completeness and applicability of the evidence in all the reviews in this suite. These have been described in the companion review on systemic pharmacological interventions for Ménière's disease (Webster 2021b) and are replicated here, as they relate to this review:

- There is a paucity of evidence about all of these interventions, despite some of them being in common use for Ménière’s disease. All the evidence we found was of very low or low certainty, showing that we are unsure of the effects of the interventions, and future research may change the effect estimates a great deal.
- We were unable to carry out many meta‐analyses. Although we identified 10 studies for inclusion, there were often differences in the actual outcomes assessed in the study, or the time points for follow‐up. Therefore, we were unable to pool the data to achieve a more precise estimate of any effect. Finally, study authors often used different ways of measuring the same outcome, which prevented data from being combined. For example, vertigo was assessed with either a global score, or a frequency score, which could not be combined, or hearing was assessed using a continuous scale or as an improvement above a certain threshold. 
- Certain outcomes were only assessed by some included studies. Many studies did not assess the impact of the disease on quality of life or tinnitus at all. Potential adverse effects of the interventions were also often poorly reported or simply not assessed. 
- We noted that unvalidated rating scales were commonly used in the studies included, particularly when looking at the global impact of treatments for vertigo. When such scales are used, it is difficult to know if they are accurately assessing the outcome, and also what size of change on this scale represents a meaningful difference in the outcome (the minimally important difference). 
- Finally, studies often failed to report clearly what treatments participants received before joining the trial, what maintenance treatment they continued on during the trial, and whether they received any additional treatments over the course of the trial. The impact of these additional treatments may be considerable, particularly for those studies with longer‐term follow‐up. Without knowing the background details of study participants (for example, the duration of their Ménière's disease, or what treatments they have tried in the past) it is difficult to identify the groups of people who may benefit from these treatments. 

### Certainty of the evidence

We used the GRADE approach to assess the certainty of the evidence in this review. The evidence identified was all low‐ or very low‐certainty, meaning that we are uncertain about the actual effect of these interventions for all of our outcomes. The main issues that affected the certainty of the evidence were the domains of study limitations, imprecision and 'other considerations' (i.e. publication bias). The different domains addressed by GRADE are considered in more detail below.

#### Study limitations/risk of bias

All the studies included in this review had at least some concerns regarding the potential for bias in the study design, conduct or reporting. Most studies did not provide a clear description of methods used to randomise participants into groups, or to conceal allocation, therefore we rated these domains at unclear risk of bias. However, we acknowledge that this may be in part due to poor reporting, rather than the actual conduct of the studies. Three studies did not appear to mask participants, study personnel or outcome assessors to the interventions used in each group, leading to a high or unclear risk of performance and detection bias (Borghei 2016; El Shafei 2020; Ul Shamas 2017). One study was at risk of attrition bias, due to poor follow‐up in the placebo group (Garduno‐Anaya 2005). We rated four further studies at unclear risk of attrition bias, as the number of participants who actually provided outcome data (or the number in whom data was imputed) was not reported (AVERTS‐1; AVERTS‐2; NCT03664674; Ul Shamas 2017). We had substantial concerns about the risk of selective reporting in this review. We rated three studies at high risk for this domain, due to incomplete reporting of outcomes that had been pre‐specified in the trial protocol/registration (AVERTS‐2; Lambert 2016), or incomplete reporting of results that precluded their inclusion in this review (Ul Shamas 2017). We had additional concerns about the conduct of two studies, leading to a high risk of 'other bias' (Borghei 2016; Ul Shamas 2017).

#### Inconsistency

We conducted few meta‐analyses in the course of this review, therefore inconsistency did not usually impact on the certainty of the evidence. For the majority of outcomes, a single study was included in the analysis. Consequently, inconsistency between studies was not of relevance. We only had one meta‐analysis where inconsistency was considered to be a concern (Analysis 1.11).

#### Indirectness

This was not a major concern for most of the outcomes. We rated down for indirectness if the majority of evidence for an outcome had come from a study where the population was not clearly defined (Borghei 2016), or if there was significant concern over the methods used to measure an outcome (for example, use of an unvalidated scoring system for vertigo, as in Lambert 2012).

#### Imprecision

Many included studies were small and, as discussed above, we were unable to carry out meta‐analyses. Therefore, the total sample size for each of our outcomes of interest was small, and reduced the certainty of the evidence. For some outcomes the resulting confidence intervals for the effect size were also extremely wide ‐ meaning that there was uncertainty over whether the intervention was beneficial or harmful. This further impacted on the certainty of the evidence.

For each analysis result, the width of the confidence interval is compared to the threshold for an important difference (details of how we selected these thresholds are given in the Methods section). If the confidence interval crosses this threshold ‐ and includes both the potential for an important benefit and the potential for a trivial effect, then the certainty of the evidence would be reduced by one level. If the confidence interval includes the possibility of *both* an important benefit and an important harm then the certainty would be reduced further. Therefore, it is important to agree on thresholds for this rating, i.e. where is the threshold, or cut‐point, between a trivial difference and a small, but important benefit or harm for each outcome? This question is difficult to answer, and requires input from people with balance disorders. As part of this review process, one of the author team (KW) joined some discussion groups for people with balance disorders, to try and obtain their views on quantifying an important and meaningful difference in treatment outcomes. However, the main theme that emerged from these discussions was that people were unable to give a specific threshold for each outcome. Instead, individuals tended to weigh up a variety of different factors when determining this threshold. The invasiveness and burden of taking the treatment would be taken into account, as well as potential side effects and the severity of their symptoms at that time. The GRADE working group would likely refer to this as a "fully contextualised approach", accounting for all aspects of the specific intervention in order to set thresholds for benefit (Zeng 2021). For this review we adopted a "minimally contextualised approach" and rated imprecision for each outcome according to specific, defined thresholds (as described in Methods). However, if the thresholds used are inappropriate then this may affect the certainty of the evidence (by a maximum of one level).

#### Other considerations

For many outcomes that were reported at 3 to < 6 months, we rated down the certainty of the evidence by one level for publication bias, due to our knowledge of unpublished studies that should have reported in this time period.

### Potential biases in the review process

As with other reviews in this suite, we made some small changes to the review process following the publication of our protocol. 

Firstly, we planned to use the Cochrane Pregnancy and Childbirth Trustworthiness Tool to assess the included studies. We had planned to exclude any study where there were concerns (as identified with this tool) from the main analyses. However, as described above, we were unable to determine whether most of the included studies would pass the screening tool, either due to a lack of reporting in the original articles, or because we were unable to contact the authors to resolve any issues. If these studies were subsequently found to have genuine concerns over research integrity then this would further undermine our confidence in the findings of the review. However, as the evidence for these interventions is almost all very low‐certainty, we considered that this would not greatly impact the findings of the review. 

We also identified that the outcome "improvement in vertigo" may not capture an important change in vertigo. Therefore, we added a sensitivity analysis for this outcome. For our main analysis we considered any improvement in vertigo, as pre‐planned. However, we also looked at whether considering "complete resolution of vertigo, or a substantial improvement in vertigo", would impact on the effect estimates. We did note that the point estimate and confidence intervals were typically shifted when using this analysis (in favour of intratympanic corticosteroids), but the evidence remained low‐certainty, therefore we cannot draw any firm conclusions from this exploratory approach. 

### Agreements and disagreements with other studies or reviews

We identified a number of published review articles that also consider the use of intratympanic corticosteroids in Ménière's disease. Many of these reviews were published some years ago, and therefore only included the oldest study in this review ‐ Garduno‐Anaya 2005. This included the previous Cochrane Review on this topic (Phillips 2011), and other reviews (Alles 2006; Doyle 2004; Hu 2009; Wright 2015). Some authors also included the study Silverstein 1998 in their analysis; however, this was excluded from our review as it was a cross‐over trial and results from the first phase of the trial were unavailable. The results of these articles are, therefore, not directly comparable with our review, due to the inclusion of different data. However, many authors highlight the sparse evidence that is available on this topic. 

More recent reviews have also included some data from Lambert 2012 and Lambert 2016 in their analyses (Chuang‐Chuang 2017; Devantier 2019; Syed 2015). Two of these reviews made similar conclusions to our own ‐ that there is a lack of solid evidence that intratympanic corticosteroids are beneficial for Ménière's disease, and the evidence is low‐certainty (Chuang‐Chuang 2017; Devantier 2019). One of the reviews was more optimistic in concluding that there are 'promising results' for intratympanic corticosteroids (Syed 2015). However, we note that this review also included the study Albu 2016 in the comparison between intratympanic dexamethasone and placebo, which we think may be an error, as all participants in this trial received intratympanic corticosteroids (with or without high‐dose betahistine). 

We identified three network meta‐analyses (NMA), which included a comparison of intratympanic corticosteroids with placebo as part of the network (Ahmadzai 2020; Cao 2019; Hao 2022). Ahmadzai 2020 and Cao 2019 only included Garduno‐Anaya 2005 for the comparison of intratympanic corticosteroids and placebo. Intratympanic corticosteroids were found to have benefits over placebo for both vertigo control and hearing in these analyses, but the confidence intervals were very wide. The certainty of the evidence was not assessed in these reviews. The NMA by Hao 2022 included data from Lambert 2012 and Lambert 2016, as well as Garduno‐Anaya 2005. This review also concluded that intratympanic corticosteroids showed beneficial effects on the management of vertigo when compared with placebo. Although GRADE was used as part of this review, the certainty of the evidence for the comparison of corticosteroids and placebo was not reported. 

The findings of our review therefore differ slightly from these NMAs, predominantly because of our use of GRADE to consider the certainty of the evidence. Whilst the numerical results may appear to favour intratympanic corticosteroids for some outcomes, a broader assessment of the certainty of the evidence makes us less sure of the overall effects. 

## Authors' conclusions

**Implications for practice**

The evidence for the use of intratympanic corticosteroids for Ménière's disease is uncertain. Methodological concerns regarding the conduct and reporting of studies in this area have led to doubts over both the efficacy and potential harms of this intervention. 

**Implications for research**

This review was conducted as part of a suite of systematic reviews regarding different interventions for Ménière's disease. Many of the conclusions below are relevant to all of these reviews and are replicated across the suite.

The lack of high‐certainty, randomised controlled trial (RCT) evidence for intratympanic corticosteroids suggests that well‐conducted studies with larger numbers of participants are required to appropriately assess the efficacy (and potential harms) of this intervention. However, there also needs to be more clarity on which outcomes studies should assess, and when and how to assess them. Vertigo is a notoriously difficult symptom to assess, and there is great variety in the methods used to record and report this symptom in the studies we have identified. 

There is a clear need for consensus on which outcomes are important to people with Ménière’s disease, so that future studies can be designed with this in mind. Development of a core outcome set would be preferable as a guide for future trials. We understand that development of a core outcome set for Ménière's disease was underway, with a project registered on the COMET website (https://www.comet-initiative.org/Studies/Details/818), but we have been unable to identify any results of this project, or ascertain whether it is ongoing. If a core outcome set is developed, this should include details on the recommended methods used to measure outcomes, ensuring that these are validated, reliable tools. Monitoring and reporting of adverse effects should be considered a routine part of any study, and should always occur ‐ this is inconsistent at present. Agreement is also needed on the appropriate times at which outcomes should be measured to adequately assess the different interventions.

Any decisions about which outcomes to measure, how to measure them and when to measure them must be made with input from people with Ménière’s disease, to ensure that the outcomes reported by trialists (and future systematic reviews) are relevant to those with the disease. 

For those considering development of a core outcome set, we would highlight that the use of the dichotomous outcome 'improvement' or 'no improvement' of vertigo may cause difficulties when interpreting the results. Individuals with Ménière's disease typically experience fluctuations in disease severity over time. Furthermore, they may have enrolled in a clinical trial at a time when their symptoms were severe. Therefore there is likely to be a natural tendency to improve over time, even for those who do not receive an intervention. The high rate of improvement in those who receive no treatment means that smaller studies are likely to be underpowered to detect a true effect of treatment. Ideally, agreement should be reached on what constitutes a *meaningful improvement* in vertigo symptoms, rather than simply considering any improvement as a positive outcome. 

Trialists should also be clear about the treatments that participants received before entry to the trial, throughout the trial, and the need for additional treatment during the course of the trial. People with Ménière's disease need to be able to understand whether interventions work in all people with the disease, or whether they might work best during certain phases of the disease ‐ perhaps as a first‐line therapy, or for people in whom other treatments have failed. 

Finally, we would re‐iterate the importance of ensuring that the results of any studies are made publicly available, to ensure that they can be incorporated into future systematic reviews and meta‐analyses in this area.  

## History

Protocol first published: Issue 12, 2021

## Appendices

### Appendix 1. AAO‐HNS definition of Ménière's disease

Definite Ménière's disease:

- Two or more spontaneous episodes of vertigo, each lasting 20 minutes to 12 hours.
- Audiometrically documented low‐ to medium‐frequency sensorineural hearing loss in one ear, defining the affected ear on at least one occasion before, during or after one of the episodes of vertigo.
- Fluctuating aural symptoms (hearing, tinnitus or fullness) in the affected ear.
- Not better accounted for by another vestibular diagnosis.

Probable Ménière's disease: 

- Two or more spontaneous episodes of vertigo, each lasting 20 minutes to 24 hours.
- Fluctuating aural symptoms (hearing, tinnitus or fullness) in the affected ear.
- Not better accounted for by another vestibular diagnosis.

Taken from Lopez‐Escamez 2015.

### Appendix 2. Search strategy

This search strategy has been designed to identify all relevant studies for a suite of reviews on various interventions for Ménière's disease. 

**Table:**

**CENTRAL (CRS) **	**Cochrane ENT Register (CRS) **	**MEDLINE (Ovid) **
1 MESH DESCRIPTOR Endolymphatic Hydrops EXPLODE ALL AND CENTRAL:TARGET 2 meniere*):AB,EH,KW,KY,MC,MH,TI,TO AND CENTRAL:TARGET 3 (endolymphatic near hydrops):AB,EH,KW,KY,MC,MH,TI,TO AND CENTRAL:TARGET 4 (labyrinth* near hydrops):AB,EH,KW,KY,MC,MH,TI,TO AND CENTRAL:TARGET 5 (labyrinth* near syndrome):AB,EH,KW,KY,MC,MH,TI,TO AND CENTRAL:TARGET 6 (aural near vertigo):AB,EH,KW,KY,MC,MH,TI,TO AND CENTRAL:TARGET 7 (labyrinth* near vertigo):AB,EH,KW,KY,MC,MH,TI,TO AND CENTRAL:TARGET 8 (cochlea near hydrops):AB,EH,KW,KY,MC,MH,TI,TO AND CENTRAL:TARGET 9 (vestibular near hydrops):AB,EH,KW,KY,MC,MH,TI,TO AND CENTRAL:TARGET 10 #1 OR #2 OR #3 OR #4 OR #5 OR #6 OR #7 OR #8 OR #9 AND CENTRAL:TARGET	1 MESH DESCRIPTOR Endolymphatic Hydrops EXPLODE ALL AND INREGISTER 2 (meniere*):AB,EH,KW,KY,MC,MH,TI,TO AND INREGISTER 3 (endolymphatic near hydrops):AB,EH,KW,KY,MC,MH,TI,TO AND INREGISTER 4 (labyrinth* near hydrops):AB,EH,KW,KY,MC,MH,TI,TO AND INREGISTER 5 (labyrinth* near syndrome):AB,EH,KW,KY,MC,MH,TI,TO AND INREGISTER 6 (aural near vertigo):AB,EH,KW,KY,MC,MH,TI,TO AND INREGISTER 7 (labyrinth* near vertigo):AB,EH,KW,KY,MC,MH,TI,TO AND INREGISTER 8 (cochlea near hydrops):AB,EH,KW,KY,MC,MH,TI,TO AND INREGISTER 9 (vestibular near hydrops):AB,EH,KW,KY,MC,MH,TI,TO AND INREGISTER 10 #1 OR #2 OR #3 OR #4 OR #5 OR #6 OR #7 OR #8 OR #9 AND INREGISTER 11 INREGISTER 12 * AND CENTRAL:TARGET 13 #11 NOT #12 14 #10 AND #13	1 exp Endolymphatic Hydrops/ 2 meniere*.ab,ti. 3 (endolymphatic adj3 hydrops).ab,ti. 4 (labyrinth* adj3 hydrops).ab,ti. 5 (labyrinth* adj3 syndrome).ab,ti. 6 (aural adj3 vertigo).ab,ti. 7 (labyrinth* adj3 vertigo).ab,ti. 8 (cochlea adj3 hydrops).ab,ti. 9 (vestibular adj3 hydrops).ab,ti. 10 1 or 2 or 3 or 4 or 5 or 6 or 7 or 8 or 9 11 randomized controlled trial.pt. 12 controlled clinical trial.pt. 13 randomized.ab. 14 placebo.ab. 15 drug therapy.fs. 16 randomly.ab. 17 trial.ab. 18 groups.ab. 19 11 or 12 or 13 or 14 or 15 or 16 or 17 or 18 20 exp animals/ not humans.sh. 21 19 not 20 22 10 and 21
**Embase (Ovid) **	**Web of Science Core Collection (Web of Knowledge) **	**Trial Registries **
1 exp Meniere disease/ 2 meniere*.ab,ti. 3 (endolymphatic adj3 hydrops).ab,ti. 4 (labyrinth* adj3 hydrops).ab,ti. 5 (labyrinth* adj3 syndrome).ab,ti. 6 (aural adj3 vertigo).ab,ti. 7 (labyrinth* adj3 vertigo).ab,ti. 8 (cochlea adj3 hydrops).ab,ti. 9 (vestibular adj3 hydrops).ab,ti. 10 1 or 2 or 3 or 4 or 5 or 6 or 7 or 8 or 9 11 Randomized controlled trial/ 12 Controlled clinical study/ 13 Random$.ti,ab. 14 randomization/ 15 intermethod comparison/ 16 placebo.ti,ab. 17 (compare or compared or comparison).ti. 18 ((evaluated or evaluate or evaluating or assessed or assess) and (compare or compared or comparing or comparison)).ab. 19 (open adj label).ti,ab. 20 ((double or single or doubly or singly) adj (blind or blinded or blindly)).ti,ab. 21 double blind procedure/ 22 parallel group$1.ti,ab. 23 (crossover or cross over).ti,ab. 24 ((assign$ or match or matched or allocation) adj5 (alternate or group$1 or intervention$1 or patient$1 or subject$1 or participant$1)).ti,ab. 25 (assigned or allocated).ti,ab. 26 (controlled adj7 (study or design or trial)).ti,ab. 27 (volunteer or volunteers).ti,ab. 28 human experiment/ 29 trial.ti. 30 11 or 12 or 13 or 14 or 15 or 16 or 17 or 18 or 19 or 20 or 21 or 22 or 23 or 24 or 25 or 26 or 27 or 28 or 29 31 (random$ adj sampl$ adj7 ("cross section$" or questionnaire$1 or survey$ or database$1)).ti,ab. 32 comparative study/ or controlled study/ 33 randomi?ed controlled.ti,ab. 34 randomly assigned.ti,ab. 35 32 or 33 or 34 36 31 not 35 37 Cross‐sectional study/ 38 randomized controlled trial/ or controlled clinical study/ or controlled study/ 39 (randomi?ed controlled or control group$1).ti,ab. 40 38 or 39 41 37 not 40 42 (((case adj control$) and random$) not randomi?ed controlled).ti,ab. 43 (Systematic review not (trial or study)).ti. 44 (nonrandom$ not random$).ti,ab. 45 Random field$.ti,ab. 46 (random cluster adj3 sampl$).ti,ab. 47 (review.ab. and review.pt.) not trial.ti. 48 we searched.ab. 49 review.ti. or review.pt. 50 48 and 49 51 update review.ab. 52 (databases adj4 searched).ab. 53 (rat or rats or mouse or mice or swine or porcine or murine or sheep or lambs or pigs or piglets or rabbit or rabbits or cat or cats or dog or dogs or cattle or bovine or monkey or monkeys or trout or marmoset$1).ti. and animal experiment/ 54 36 or 41 or 42 or 43 or 44 or 45 or 46 or 47 or 50 or 51 or 52 55 30 not 54 56 10 and 55	# 12 #11 AND #10 Indexes=SCI‐EXPANDED, SSCI, A&HCI, CPCI‐S, CPCI‐SSH, BKCI‐S, BKCI‐SSH, ESCI, CCR‐EXPANDED, IC Timespan=All years # 11 TOPIC: ((randomised OR randomized OR randomisation OR randomisation OR placebo* OR (random* AND (allocat* OR assign*) ) OR (blind* AND (single OR double OR treble OR triple) ))) Indexes=SCI‐EXPANDED, SSCI, A&HCI, CPCI‐S, CPCI‐SSH, BKCI‐S, BKCI‐SSH, ESCI, CCR‐EXPANDED, IC Timespan=All years # 10 #9 OR #8 OR #7 OR #6 OR #5 OR #4 OR #3 OR #2 OR #1 Indexes=SCI‐EXPANDED, SSCI, A&HCI, CPCI‐S, CPCI‐SSH, BKCI‐S, BKCI‐SSH, ESCI, CCR‐EXPANDED, IC Timespan=All years # 9 TOPIC: (vestibular NEAR/3 hydrops) Indexes=SCI‐EXPANDED, SSCI, A&HCI, CPCI‐S, CPCI‐SSH, BKCI‐S, BKCI‐SSH, ESCI, CCR‐EXPANDED, IC Timespan=All years # 8 TOPIC: (cochlea NEAR/3 hydrops) Indexes=SCI‐EXPANDED, SSCI, A&HCI, CPCI‐S, CPCI‐SSH, BKCI‐S, BKCI‐SSH, ESCI, CCR‐EXPANDED, IC Timespan=All years # 7 TOPIC: (labyrinth* NEAR/3 vertigo) Indexes=SCI‐EXPANDED, SSCI, A&HCI, CPCI‐S, CPCI‐SSH, BKCI‐S, BKCI‐SSH, ESCI, CCR‐EXPANDED, IC Timespan=All years # 6 TOPIC: (labyrinth* adj3 vertigo) Indexes=SCI‐EXPANDED, SSCI, A&HCI, CPCI‐S, CPCI‐SSH, BKCI‐S, BKCI‐SSH, ESCI, CCR‐EXPANDED, IC Timespan=All years # 5 TOPIC: (aural NEAR/3 vertigo) Indexes=SCI‐EXPANDED, SSCI, A&HCI, CPCI‐S, CPCI‐SSH, BKCI‐S, BKCI‐SSH, ESCI, CCR‐EXPANDED, IC Timespan=All years # 4 TOPIC: (labyrinth* NEAR/3 syndrome) Indexes=SCI‐EXPANDED, SSCI, A&HCI, CPCI‐S, CPCI‐SSH, BKCI‐S, BKCI‐SSH, ESCI, CCR‐EXPANDED, IC Timespan=All years # 3 TOPIC: (labyrinth* NEAR/3 hydrops) Indexes=SCI‐EXPANDED, SSCI, A&HCI, CPCI‐S, CPCI‐SSH, BKCI‐S, BKCI‐SSH, ESCI, CCR‐EXPANDED, IC Timespan=All years # 2 TOPIC: (endolymphatic NEAR/3 hydrops) Indexes=SCI‐EXPANDED, SSCI, A&HCI, CPCI‐S, CPCI‐SSH, BKCI‐S, BKCI‐SSH, ESCI, CCR‐EXPANDED, IC Timespan=All years # 1 TOPIC: (meniere*) Indexes=SCI‐EXPANDED, SSCI, A&HCI, CPCI‐S, CPCI‐SSH, BKCI‐S, BKCI‐SSH, ESCI, CCR‐EXPANDED, IC Timespan=All years	Clinicaltrials.gov menieres or meniere or meniere's | Interventional Studies ICTRP Meniere*

The date restrictions applied to the September 2022 update searches were as follows:

**Table:**

** **	**September 2022 update**
**CENTRAL**	15/09/2021_TO_14/09/2022:CRSINCENTRAL AND CENTRAL:TARGET
**ENT register**	No new records added to register since search was run
**Medline**	23 limit 22 to ed=20210915‐20220914 24 limit 22 to dt=20210915‐20220914 25 23 or 24
**Embase**	57 limit 56 to dd=20210915‐20220914
**Web of Science**	Timespan: 2021‐09‐15 to 2022‐09‐14 (Index Date)
**Clinicaltrials.gov**	First posted from 09/15/2021 to 09/14/2022
**ICTRP**	Date of registration after 15/09/2021
**Google Scholar**	Year: 2021 or 2022

### Appendix 3. Trustworthiness Screening Tool

This screening tool has been developed by Cochrane Pregnancy and Childbirth. It includes a set of predefined criteria to select studies that, based on available information, are deemed to be sufficiently trustworthy to be included in the analysis. These criteria are:

**Research governance**

- Are there any retraction notices or expressions of concern listed on the Retraction Watch Database relating to this study?
- Was the study prospectively registered (for those studies published after 2010)? If not, was there a plausible reason?
- When requested, did the trial authors provide/share the protocol and/or ethics approval letter?
- Did the trial authors engage in communication with the Cochrane Review authors within the agreed timelines?
- Did the trial authors provide IPD data upon request? If not, was there a plausible reason?

**Baseline characteristics**

- Is the study free from characteristics of the study participants that appear too similar (e.g. distribution of the mean (SD) excessively narrow or excessively wide, as noted by Carlisle 2017)?

**Feasibility**

- Is the study free from characteristics that could be implausible? (e.g. large numbers of women with a rare condition (such as severe cholestasis in pregnancy) recruited within 12 months);
- In cases with (close to) zero losses to follow‐up, is there a plausible explanation?

**Results**

- Is the study free from results that could be implausible? (e.g. massive risk reduction for main outcomes with small sample size)?
- Do the numbers randomised to each group suggest that adequate randomisation methods were used (e.g. is the study free from issues such as unexpectedly even numbers of women 'randomised' including a mismatch between the numbers and the methods, if the authors say 'no blocking was used' but still end up with equal numbers, or if the authors say they used 'blocks of 4' but the final numbers differ by 6)?

Studies assessed as being potentially 'high risk' will be not be included in the review. Where a study is classified as 'high risk' for one or more of the above criteria we will attempt to contact the study authors to address any possible lack of information/concerns. If adequate information remains unavailable, the study will remain in 'awaiting classification' and the reasons and communications with the author (or lack of) described in detail.

The process is described in full in Figure 2. 

## Data and analyses

**Comparison 1:** Intratympanic corticosteroids versus placebo/no treatment

Outcome or subgroup title	No. of studies	No. of participants	Statistical method	Effect size
1.1 Improvement in vertigo frequency	2		Risk Ratio (M‐H, Random, 95% CI)	Subtotals only
1.1.1 6 to ≤ 12 months	2	60	Risk Ratio (M‐H, Random, 95% CI)	1.00 [0.92, 1.10]
1.1.2 > 12 months	2	58	Risk Ratio (M‐H, Random, 95% CI)	1.03 [0.87, 1.23]
1.2 Improvement in vertigo frequency: sensitivity analysis for complete/substantial improvement	3		Risk Ratio (M‐H, Random, 95% CI)	Subtotals only
1.2.1 3 to < 6 months	1	36	Risk Ratio (M‐H, Random, 95% CI)	1.15 [0.81, 1.64]
1.2.2 6 to ≤ 12 months	3	96	Risk Ratio (M‐H, Random, 95% CI)	1.23 [1.01, 1.48]
1.2.3 > 12 months	2	58	Risk Ratio (M‐H, Random, 95% CI)	1.30 [1.02, 1.65]
1.3 Change in vertigo (global score) at 3 to < 6 months	1	44	Mean Difference (IV, Random, 95% CI)	‐0.13 [‐0.42, 0.16]
1.4 Change in vertigo frequency at 3 to < 6 months	3		Mean Difference (IV, Random, 95% CI)	Subtotals only
1.4.1 3 to < 6 months	3		Mean Difference (IV, Random, 95% CI)	‐0.05 [‐0.07, ‐0.02]
1.5 Change in vertigo frequency at 6 to < 12 months and > 12 months	1		Mean Difference (IV, Random, 95% CI)	Subtotals only
1.5.1 6 to ≤ 12 months	1	20	Mean Difference (IV, Random, 95% CI)	‐0.10 [‐0.79, 0.59]
1.5.2 > 12 months	1	18	Mean Difference (IV, Random, 95% CI)	‐0.07 [‐0.84, 0.70]
1.6 Serious adverse events	4	500	Risk Ratio (M‐H, Random, 95% CI)	0.64 [0.22, 1.85]
1.7 Disease‐specific health‐related quality of life	1		Mean Difference (IV, Random, 95% CI)	Subtotals only
1.7.1 6 to ≤ 12 months	1	20	Mean Difference (IV, Random, 95% CI)	‐0.38 [‐1.56, 0.80]
1.7.2 > 12 months	1	18	Mean Difference (IV, Random, 95% CI)	‐0.45 [‐2.03, 1.13]
1.8 Change in hearing: continuous data	1		Mean Difference (IV, Random, 95% CI)	Subtotals only
1.8.1 6 to ≤ 12 months	1	20	Mean Difference (IV, Random, 95% CI)	‐4.95 [‐16.50, 6.60]
1.8.2 > 12 months	1	18	Mean Difference (IV, Random, 95% CI)	‐2.84 [‐9.61, 3.93]
1.9 Improvement in hearing: dichotomous data	4		Risk Ratio (M‐H, Random, 95% CI)	Subtotals only
1.9.1 3 to < 6 months	2	184	Risk Ratio (M‐H, Random, 95% CI)	0.45 [0.18, 1.15]
1.9.2 6 to ≤ 12 months	2	76	Risk Ratio (M‐H, Random, 95% CI)	0.73 [0.45, 1.17]
1.9.3 > 12 months	2	58	Risk Ratio (M‐H, Random, 95% CI)	0.79 [0.46, 1.35]
1.10 Change in tinnitus	2		Mean Difference (IV, Random, 95% CI)	Subtotals only
1.10.1 3 to < 6 months	1	44	Mean Difference (IV, Random, 95% CI)	‐9.69 [‐20.28, 0.89]
1.10.2 6 to ≤ 12 months	1	20	Mean Difference (IV, Random, 95% CI)	‐1.12 [‐24.75, 22.51]
1.10.3 > 12 months	1	18	Mean Difference (IV, Random, 95% CI)	6.60 [‐7.79, 20.99]
1.11 Other adverse effects	4		Peto Odds Ratio (Peto, Fixed, 95% CI)	Subtotals only
1.11.1 Persistent tympanic membrane perforation	3	320	Peto Odds Ratio (Peto, Fixed, 95% CI)	5.71 [1.56, 20.96]
1.11.2 Ear pain	4	500	Peto Odds Ratio (Peto, Fixed, 95% CI)	1.19 [0.47, 3.04]
1.11.3 Post‐injection vertigo	3	346	Peto Odds Ratio (Peto, Fixed, 95% CI)	1.78 [0.65, 4.87]
1.11.4 New onset, permanent and total hearing loss in the affected ear	2	172	Peto Odds Ratio (Peto, Fixed, 95% CI)	3.47 [0.02, 486.20]
1.11.5 New onset of tinnitus in the affected ear	2	302	Peto Odds Ratio (Peto, Fixed, 95% CI)	1.09 [0.30, 3.91]

## Characteristics of studies

### Characteristics of included studies [ordered by study ID]

**Table:** AVERTS‐1

***Study characteristics***		
Methods	Phase 3, parallel‐group RCT with a single dose of OTO‐104 and 3 months of follow‐up	
Participants	**Setting:** Multicentre trial based in the USA **Sample size:** **Number randomised:** 165 participants **Number completed:** not reported **Participant baseline characteristics** No characteristics are reported. Assumed definite, unilateral Ménière's disease, due to inclusion criteria **Inclusion criteria:** Aged 18 to 85 years. Diagnosis of unilateral Ménière's disease by AAO‐HNS 1995 criteria and active vertigo for the 2 months prior to the study lead‐in period. Asymmetric sensorineural hearing loss. Agrees to maintain their current treatments for Ménière's disease while on‐study. **Exclusion criteria:** Pregnancy or lactation. History of immunodeficiency disease. History of previous endolymphatic sac surgery. History of previous use of intratympanic (IT) gentamicin in the affected ear. History of tympanostomy tubes with evidence of perforation or lack of closure. Previous adverse reaction to IT injection of steroids. Use of an investigational drug or device in the 3 months prior to screening. Previously been randomized to a trial of OTO‐104. **Diagnosis of Ménière's disease:** Diagnosed with Ménière's disease according to the AAO‐HNS 1995 guidelines. Presumed definite disease, as inclusion criteria state the need for documented sensorineural hearing loss.	
Interventions	**Intervention (number randomised and completed not reported)** Single intratympanic injection of 12 mg OTO‐104 **Comparator (number randomised and completed not reported)** Single intratympanic injection of placebo **Background interventions administered to all participants** Not reported	
Outcomes	**Primary outcomes relevant to this review:** **Improvement in vertigo** Not reported **Change in vertigo** Assessed with number of definitive vertigo days per month at 3 months; not fully reported **Serious adverse events** Assessed, according to trial registry site, but not reported **Secondary outcomes relevant to this review:** **Disease‐specific health‐related quality of life** Trial registry site states that "impact of vertigo experience on daily activities" will be assessed, but this is not reported **Hearing** Trial registry states that this will be assessed with audiometry at 3 months but no data are reported **Tinnitus ** Not apparently assessed or reported **Other adverse effects** Assessed, according to trial registry site, but not reported **Other outcomes reported in the study:** Otoscopic examinations, but these results are not reported	
Funding sources	Sponsored by Otonomy Inc.	
Declarations of interest	This trial remains unpublished, but was funded by Otonomy	
Notes	This trial was completed in July 2017. The results are currently unpublished, and no results are posted on the trial registry site. We have contacted the company, but they have been unable to supply us with the results of this trial. The only data available are published in a press release on the company website.	
***Risk of bias***		
**Bias**	**Authors' judgement**	**Support for judgement**
Random sequence generation (selection bias)	Unclear risk	Comment: This trial remains unpublished. No information is available to assess the risk of bias.
Allocation concealment (selection bias)	Unclear risk	Comment: This trial remains unpublished. No information is available to assess the risk of bias.
Blinding of participants and personnel (performance bias) All outcomes	Unclear risk	Comment: This trial remains unpublished. No information is available to assess the risk of bias.
Blinding of outcome assessment (detection bias) All outcomes	Unclear risk	Comment: This trial remains unpublished. No information is available to assess the risk of bias.
Incomplete outcome data (attrition bias) All outcomes	Unclear risk	Comment: This trial remains unpublished. No information is available to assess the risk of bias.
Selective reporting (reporting bias)	Unclear risk	Comment: This trial remains unpublished. No information is available to assess the risk of bias.
Other bias	Unclear risk	Comment: This trial remains unpublished. No information is available to assess the risk of bias.

**Table:** AVERTS‐2

***Study characteristics***		
Methods	Parallel‐group, double‐blind phase 3 RCT with a single administration of intervention and 12 weeks of follow‐up. This study was prematurely terminated due to negative efficacy results from another (similar) trial, run by the same company.	
Participants	**Setting:** Multicentre trial based in Europe, recruitment from March 2016 to August 2017; 49 sites in the UK, 8 sites in Belgium, 9 sites in France, 28 sites in Germany, 18 sites in Italy and 62 sites in Poland **Sample size:** **Number randomised:** 176 participants, 174 participants received the study drug **Number completed:** 112 participants **Participant baseline characteristics** **Age:** OTO‐104 group: mean 51.8 years (SD 12.13 years) Placebo group: mean 52.6 years (SD 13.24 years) **Gender:** OTO‐104 group: 42 males (49%): 44 females (51%) Placebo group: mean 40 males (45%): 48 females (55%) **Probable/definite Ménière's disease:** Presumed definite disease, as AAO‐HNS 1995 criteria were used, and inclusion criteria state the need for documented sensorineural hearing loss **Duration of disease:** Not reported **Attack frequency at baseline:** Not reported **Hearing loss at baseline:** Not reported **Measure of tinnitus at baseline:** Not reported **Number of participants with bilateral disease:** None (inclusion criterion of unilateral disease) **Inclusion criteria:** Diagnosis of unilateral Ménière's disease by AAO‐HNS 1995 criteria and reports active vertigo for the 2 months prior to the study lead‐in period. Active vertigo during the lead‐in period (not defined further on trial registry site). Documented asymmetric sensorineural hearing loss. Agrees to maintain their current treatments for Ménière's disease while on‐study. **Exclusion criteria:** Pregnancy or lactation. History of immunodeficiency disease. History of previous endolymphatic sac surgery. History of previous use of intratympanic (IT) gentamicin in the affected ear. History of tympanostomy tubes with evidence of perforation or lack of closure. Previous adverse reaction to IT injection of steroids. Use of an investigational drug or device in the 3 months prior to screening. Previously been randomised to a trial of OTO‐104. **Diagnosis of Ménière's disease:** Diagnosed with Ménière's disease according to the AAO‐HNS 1995 guidelines. Presumed definite disease, as inclusion criteria state the need for documented sensorineural hearing loss.	
Interventions	**Intervention (n = 86 randomised, n = 53 completed)** Single intratympanic injection of 12 mg OTO‐104: 0.2 mL of a 6% suspension of dexamethasone in poloxamer 407 aqueous solution **Comparator (n = 88 randomised, n = 59 completed)** Single intratympanic injection of placebo: 0.2 mL poloxamer 407 aqueous solution **Background interventions administered to all participants** Participants were recommended to continue on the medications they were on prior to the start of the study, which may include betahistine, diuretics and/or a low salt diet	
Outcomes	**Primary outcomes relevant to this review:** **Improvement in vertigo** Not reported **Change in vertigo** Assessed with number of definitive vertigo days per month at 12 weeks **Serious adverse events** Assessed and reported **Secondary outcomes relevant to this review:** **Disease‐specific health‐related quality of life** Trial registry site states that "impact of vertigo experience on daily activities" will be assessed, but this is not reported **Hearing** Trial registry states that this will be assessed with audiometry at 3 months but no data are reported **Tinnitus ** Not apparently assessed of reported **Other adverse effects** Some adverse effects were assessed and reported; tympanic membrane perforation is not reported **Other outcomes reported in the study:** Planned to assess the change in the condition of the ear from baseline, as assessed with otoscopy, but these results are not reported	
Funding sources	Sponsored by Otonomy Inc.	
Declarations of interest	Not reported in the conference abstract, but noted that one author is an employee of Autonomy Inc. Trial funded by Otonomy.	
Notes	Information on this trial was identified from a conference abstract and trial registration details only. The study was prematurely terminated. Trial registration site states that this was because of the "negative efficacy results from a recently completed phase 3 study". **Research integrity checklist:** No retractions/expressions of concern were identified, but the study remains unpublished Limited baseline characteristics of trial participants are reported, but no concerns based on the available data The study was terminated by the sponsor, therefore there is substantial dropout from both groups No implausible results from the (limited) data reported No concerns over the adequacy of randomisation	
***Risk of bias***		
**Bias**	**Authors' judgement**	**Support for judgement**
Random sequence generation (selection bias)	Unclear risk	Comment: no information is provided regarding methods of randomisation.
Allocation concealment (selection bias)	Unclear risk	Comment: no information is provided on the process for randomisation and any methods for concealment of allocation.
Blinding of participants and personnel (performance bias) All outcomes	Low risk	Quote: "A treatment syringe (OTO‐104 or placebo) was pre‐loaded by an unblinded person. Each syringe was prepared to prevent visualization of syringe contents by all other study staff through the use of a syringe overlabel. Any interaction with subjects with regard to the collection, review or discussion of study assessments, with the exception of otoscopic exams, was done by the study coordinator, audiologist or someone other than the person who prepared the syringe and the physician who administered" Comment: it was impossible to blind the person who actually delivered the treatment (due to the different appearance of the study drug), but this person was not involved in follow‐up of participants, therefore unlikely to cause a risk of performance bias.
Blinding of outcome assessment (detection bias) All outcomes	Low risk	Quote: "A treatment syringe (OTO‐104 or placebo) was pre‐loaded by an unblinded person. Each syringe was prepared to prevent visualization of syringe contents by all other study staff through the use of a syringe overlabel. Any interaction with subjects with regard to the collection, review or discussion of study assessments, with the exception of otoscopic exams, was done by the study coordinator, audiologist or someone other than the person who prepared the syringe and the physician who administered". Comment: some outcomes were reported by blinded participants. Trial registry states that interaction with participants for collection of outcome data was performed by blinded study staff.
Incomplete outcome data (attrition bias) All outcomes	Unclear risk	Comment: analysis of primary outcome states that 174 participants were included. However, the authors also state that 34 participants in the OTO‐104 group and 29 participants in the placebo group did not complete the trial as the study was terminated. It is unclear whether they all completed the 3‐month time frame for reporting of the primary outcome, or whether data have been imputed for this analysis due to missing outcome data.
Selective reporting (reporting bias)	High risk	Comment: the ClinicalTrials.gov trial registry entry states that hearing will be assessed, as well as quality of life and condition of the ear. None of these outcomes are reported in the EU CTR trial registry records. Tympanic membrane perforation is not reported, despite this being a specific concern with this intervention.
Other bias	Low risk	Comment: no other concerns noted.

**Table:** Borghei 2016

***Study characteristics***		
Methods	Parallel‐group, single‐blinded RCT with 3 months of treatment and follow‐up for a further 9 months (12 months duration of follow‐up in total)	
Participants	**Setting: ** Single‐centre study conducted at Tehran University of Medical Sciences, Iran from January 2013 to January 2014 **Sample size:** **Number randomised:** 36 participants **Number completed:** 36 participants **Participant baseline characteristics** **Age:** given for whole cohort: 10 to 19 years: 2 participants (5.5%) 20 to 29 years: 4 participants (11.1%) 30 to 39 years: 13 participants (36.1%) 40 to 49 years: 8 participants (22.2%) 50 to 59 years: 7 participants (19.4%) 60 to 69 years: 2 participants (5.5%) **Gender:** given for whole cohort Male: 16 (44.4%) Female: 20 (55.5%) **Probable/definite Ménière's disease:** not reported **Duration of disease:** given for whole cohort 0 to 1 years: 12 (33.3%) 1 to 2 years: 8 (22.2%) 2 to 3 years: 6 (16.6%) 3 to 4 years: 3 (8.3%) 4 to 5 years: 3 (8.3%) > 5 years: 4 (11.1%) **Attack frequency at baseline:** not reported **Hearing loss at baseline:** not reported **Measure of tinnitus at baseline:** not reported **Number of participants with bilateral disease:** not reported **Inclusion criteria:** Not fully reported. Article states "all patients with intractable Meniere's Disease". Study appears to have recruited individuals in whom a low‐sodium diet and betahistine plus triamterene‐H (plus "anti‐vertigo/antiemetics") have failed to control symptoms over a 2‐month period. **Exclusion criteria:** Those patients who initially responded to a low salt diet and betahistine were not recruited for the actual RCT. No other information on exclusion criteria. **Diagnosis of Ménière's disease:** No details reported. Article states "all patients with intractable Meniere's Disease".	
Interventions	**Intervention (n = 18 randomised, n = 18 completed)** Dexamethasone group. All participants underwent ventilation tube placement. Dexamethasone drops (dosage not specified) were instilled by the participants themselves every other day. Participants were instructed to be in a supine position with the head rotated 45 degrees to the contralateral side, keeping the treated ear up. Participants were instructed not to swallow for 20 minutes to allow enough time for the medication to be absorbed. Treatment was continued for 3 months. **Comparator (n = 18 randomised, n = 18 completed)** Placebo group. All participants underwent ventilation tube placement. Placebo drops (contents not reported) were used with the same regime as the dexamethasone group, administered by the participants themselves on alternate days for 3 months. Adherence to interventions was not reported. Efficacy of blinding was not assessed. **Background interventions administered to all participants** All participants were advised to have a low‐sodium diet (≤ 1500 mg sodium per day) and were prescribed betahistine plus triamterene‐H (dose not stated) in the 2 months preceding randomisation. It is unclear whether these treatments were also continued during the course of the study. Author also state that "On occasion, ant vertigo/antiemetic agents were used".	
Outcomes	**Primary outcomes relevant to this review:** **Improvement in vertigo** Improvement appears to have been assessed using criteria that considered both hearing and vertigo, using a modification of the AAOO 1972 classes of results of treatment. Classes were as follows: A: at least 10 dB improvement in comparison to the best hearing threshold before treatment and absence of recurrent vertigo B1: no change in hearing threshold in comparison to the best hearing threshold before treatment and absence of recurrent vertigo B2: hearing threshold between the best and the worst hearing threshold before treatment and absence of recurrent vertigo C: hearing threshold equal to the worst hearing threshold before treatment and absence of recurrent vertigo D: hearing threshold worse than the worst hearing threshold before treatment and absence of recurrent vertigo E: A or B criteria and recurrent vertigo F: C or D criteria and recurrent vertigo Those whose vertigo has improved were therefore scored as A, B1, B2, C or D. It is unclear how severe vertigo symptoms were at trial entry, but we assume that all participants had vertigo. **Change in vertigo** Not reported **Serious adverse events** Not fully reported; narrative description of one serious adverse event only **Secondary outcomes relevant to this review:** **Disease‐specific health‐related quality of life** Not reported **Hearing** Reported according to the same scale as that used for vertigo (above), as a dichotomous outcome: improved/unchanged/worsened. Hearing was assessed with auditory tests at 500 Hz, 1000 Hz, 2000 Hz and 4000 Hz. A 10 dB change in at least 2 frequencies was considered to be significant. **Tinnitus ** Not assessed with a validated questionnaire **Other adverse effects** Not fully reported; narrative description of one adverse event only **Other outcomes reported in the study:** Aural fullness	
Funding sources	Financial support from Tehran University of Medical Sciences	
Declarations of interest	The authors declare no conflicts of interest	
Notes	**Research integrity checklist:** No retractions or expressions of concern were identified No published protocol or trial registration was identified, although the study was published after 2010 Baseline characteristics of the 2 groups were not reported separately, therefore we are unable to establish whether these were excessively similar No participants were reported to have dropped out during the trial No implausible results are reported The authors state that block randomisation was used, which may account for the equal number of participants in each group; however, limited details are available on the randomisation process	
***Risk of bias***		
**Bias**	**Authors' judgement**	**Support for judgement**
Random sequence generation (selection bias)	Unclear risk	Quote: "Patients were randomly allocated to two groups of 18‐patients using block randomization." Comment: no further details provided regarding methods used for randomisation, size of blocks etc.
Allocation concealment (selection bias)	Unclear risk	Quote: "Patients were randomly allocated to two groups of 18‐patients using block randomization." Comment: no further details provided regarding methods used to ensure allocation was concealed.
Blinding of participants and personnel (performance bias) All outcomes	High risk	Quote: "All patients were unaware of the type of treatment and were blinded, but surgeon was not blinded." Comment: although participants were blinded, other study personnel were not blinded to the intervention therefore could have influenced performance over the course of the study.
Blinding of outcome assessment (detection bias) All outcomes	Unclear risk	Comment: limited information is available regarding the methods of outcome assessment, including for the vertigo outcomes. Although participants were blinded to their allocation, surgeons were not blinded. It is unclear who would have assessed vertigo as being "improved" or not, and how this was assessed, therefore we have rated this domain as unclear risk.
Incomplete outcome data (attrition bias) All outcomes	Low risk	Comment: full follow‐up is reported.
Selective reporting (reporting bias)	Unclear risk	Comment: no protocol was identified to enable this domain to be assessed fully.
Other bias	High risk	Comment: limited details on how outcomes were assessed. Unclear whether "presence of vertigo" means any vertigo ‐ if so, then this study has overall a very high rate of success in both the treatment and placebo arms. Insufficient detail on the population included; not clear how participants were diagnosed with Ménière's disease. The age distribution of participants in this trial seems unusual, with the majority of participants in the age range 30 to 39. This is not typical of Ménière's disease, therefore there may be concerns over the diagnostic criteria used.

**Table:** El Shafei 2020

***Study characteristics***		
Methods	Parallel‐group, 3‐arm RCT with 3 weeks duration of treatment and follow‐up for a total of 18 months Groups included: intratympanic dexamethasone injections, intratympanic dexamethasone drops administered via injection through a ventilation tube and saline administered via injection through a ventilation tube. For this review we are only able to include the comparison of dexamethasone drops through a ventilation tube versus saline through a ventilation tube. Data on the third arm of the study are not included below.	
Participants	**Setting:** ENT department of a University Hospital in Egypt **Sample size: ** **Number randomised:** 40 participants **Number completed:** 40 participants **Participant baseline characteristics** **Age:** Dexamethasone group: mean 42.4 years (SD 7.6) Placebo group: mean 41.1 years (SD 7.1) **Gender:** Dexamethasone group: 9 male (45%); 11 female (55%) Placebo group: 7 male (35%); 13 female (65%) **Probable/definite Ménière's disease:** Unclear, not stated explicitly in the article **Duration of disease:** Dexamethasone group 1 year: 10 participants (presumed to mean up to 1 year) 5 years: 10 participants (presumed to mean > 1 but ≤ 5 years) Placebo group 1 year: 11 participants (presumed to mean up to 1 year) 5 years: 9 participants (presumed to mean > 1 but ≤ 5 years) **Attack frequency at baseline:** Not reported **Hearing loss at baseline:** Dexamethasone group: median 55 dB (IQR 13.8) Placebo group: median 52.5 dB (IQR 10) **Measure of tinnitus at baseline:** Not reported **Number of participants with bilateral disease:** Not reported; participants presumed to have unilateral disease **Inclusion criteria:** Diagnosed with Ménière's disease according to the AAO‐HNS 1995 guidelines. Prior to enrollment, all patients had previously received medical treatment for at least 6 months and underwent full audiologic, otoneurologic and videonystagmographic (VNG) examinations. **Exclusion criteria:** Magnetic resonance imaging was done to rule out any retrocochlear disorders. **Diagnosis of Ménière's disease:** Diagnosed with Ménière's disease according to the AAO‐HNS 1995 guidelines. Unclear if participants had probable or definite disease.	
Interventions	**Intervention (n = 20 randomised, n = 20 completed)** Dexamethasone group. A tympanostomy tube was inserted in the affected ear, in the anterior‐inferior part of the tympanic membrane under local or general anaesthesia. Participants received intratympanic 4 mg/mL (volume not stated) dexamethasone injection through the tympanostomy tube every week for 3 weeks. **Comparator (n = 20 randomised, n = 20 completed)** Placebo group. A tympanostomy tube was inserted as above, and participants received saline injections through the tube every week for 3 weeks. **Background interventions administered to all participants:** All participants had a ventilation tube inserted	
Outcomes	**Primary outcomes relevant to this review:** **Improvement in vertigo** Improvement in vertigo frequency was assessed using the class of vertigo control, as defined by the AAO‐HNS 1995 **Change in vertigo** Not reported **Serious adverse events** Not reported **Secondary outcomes relevant to this review:** **Disease‐specific health‐related quality of life** Not reported **Hearing** Reported as the number of people whose hearing improved, as assessed using a pure tone audiogram at 0.5 Hz, 1 Hz, 2 Hz and 4 kHz; improvement was defined as a hearing gain of > 10 dB **Tinnitus ** Not reported **Other adverse effects** Not reported **Other outcomes reported in the study:** Correlation between duration of Ménière's disease symptoms and recovery	
Funding sources	"Funding statement: none declared" (we assume no funding was received for this study)	
Declarations of interest	"Competing interested: none declared" (we assume authors do not have any conflicts of interest)	
Notes	**Research integrity checklist:** No retractions or expressions of concern were identified No published protocol or trial registration was identified, although the study was published after 2010 Baseline characteristics of the 2 groups do not appear to be excessively similar No loss to follow‐up was reported and no reason is given for this No implausible results were identified Equal numbers of participants were randomised to each group, without mention of block randomisation	
***Risk of bias***		
**Bias**	**Authors' judgement**	**Support for judgement**
Random sequence generation (selection bias)	Low risk	Quote: "The patients were randomly divided through computer into three groups". Comment: computerised randomisation, which should be adequate.
Allocation concealment (selection bias)	Unclear risk	Comment: no information is provided regarding concealment of allocation.
Blinding of participants and personnel (performance bias) All outcomes	High risk	Comment: no description of blinding, therefore we assume this was an open‐label trial. Although the 2 interventions included for this review both involved placement of a ventilation tube, the third intervention did not. This indicates that participants were likely to have been aware of the group allocation, as they would have known whether or not a ventilation tube had been placed.
Blinding of outcome assessment (detection bias) All outcomes	High risk	Comment: there is no statement that outcome assessors were blinded to group allocation. Some outcomes will have been rated by un‐blinded participants.
Incomplete outcome data (attrition bias) All outcomes	Low risk	Comment: complete follow‐up is reported.
Selective reporting (reporting bias)	Unclear risk	Comment: no protocol is available to assess the potential for selective reporting.
Other bias	Low risk	Comment: no other concerns were identified.

**Table:** Garduno‐Anaya 2005

***Study characteristics***		
Methods	Parallel‐group, double‐blind, randomised controlled trial with 5 days duration of treatment and up to 2 years duration of follow‐up. Note that if participants in the control group "failed treatment" at any point during the trial then alternate interventions were offered and they were excluded from further follow‐up. It is not clear how this was determined.	
Participants	**Setting:** Department of Neurotology of the National Institute of Neurology and Neurosurgery in Mexico City, Mexico. Study conducted from November 2000 until July 2003. **Sample size:** **Number randomised:** 24, but 2 declined to participate **Number completed:** 18 **Participant baseline characteristics** **Age:** Dexamethasone group: mean 49 years (SD 16.76) Placebo group: mean 50.91 years (SD 14.87) **Gender:** Dexamethasone group: 3 male (27%): 8 female (73%) Placebo group: 3 male (27%): 8 female (73%) **Probable/definite Ménière's disease:** All participants had definite Ménière's disease **Duration of disease:** Not reported **Attack frequency at baseline:** Dexamethasone group: mean 0.87 attacks per month (SD 0.61) Placebo group: mean 1.03 attacks per month (SD 0.80) **Hearing loss at baseline:** Dexamethasone group: mean hearing threshold using PTA 55.73 dB (SD 18.24) Placebo group: mean hearing threshold using PTA 56.64 dB (SD 13.55) **Measure of tinnitus at baseline:** Not reported **Number of participants with bilateral disease:** All participants had unilateral disease **Inclusion criteria:** Patients with definite Ménière's disease, according to the AAO‐HNS 1995, aged over 18 years. Failed to respond to conventional medical therapy (caffeine and salt restriction < 1500 mg per day, vasodilator and diuretic) when administered for 6 months **Exclusion criteria:** Declined to participate. Bilateral disease. Response to conventional medical therapy. Previous treatment with steroids or surgery for Ménière's disease. **Diagnosis of Ménière's disease:** Diagnosed with definite Ménière's disease according to the AAO‐HNS 1995 guidelines	
Interventions	**Intervention (n = 11 randomised, n = 11 completed)** Dexamethasone (4 mg/mL) was administered slowly through the tympanic membrane with a 22 G needle and a 3 mL syringe, after application of local anaesthetic. The quantity varied from 0.5 mL to 0.8 mL, with a mean of 0.5 mL. The patient was instructed to lie supine with the treated ear up for 1 hour, to keep their head still, swallow as little as possible and not to talk. The procedure was repeated daily for a total of 5 consecutive days. **Comparator (n = 11 randomised, n = 7 completed)** Participants underwent the same procedure as outlined above, but saline solution was used. No further details about the placebo were provided. **Background interventions administered to all participants** None reported. Before the study, all participants had failed to respond to conventional medical therapy. It is unclear whether these medications were also continued for the duration of the trial.	
Outcomes	**Primary outcomes relevant to this review:** **Improvement in vertigo** Improvement in vertigo frequency was assessed using the class of vertigo control, as defined by the AAO‐HNS 1995 **Change in vertigo** Change in vertigo frequency was assessed using the number of "definitive episodes of vertigo" per month. Not clear what constitutes a definitive episode. We assume this means an episode of 20 minutes or longer, as described by the AAO‐HNS 1995, although it is not clear how patients reported their episodes, and whether this was done contemporaneously. **Serious adverse events** Not fully reported and unclear how these were assessed; narrative description that "we did not have any complications" **Secondary outcomes relevant to this review:** **Disease‐specific health‐related quality of life** Participants completed both the Functional Level Scale and the Dizziness Handicap Inventory. As data for the DHI were only reported graphically (with medians and IQR) and because there appears to be a discrepancy in the data between the graph and the text, data from the FLS were used for analysis. **Hearing** Assessed with average hearing threshold from a pure tone audiogram in dB (frequencies were not reported). Also reported on number of participants who improved (≥ 10dB) at 24 months follow‐up. **Tinnitus ** Assessed with the Tinnitus Handicap Inventory (THI). Data were predominantly reported as medians and IQR, except for the mean endpoint score at 24 months for each group. For analysis, means and standard deviations were estimated from the data reported using methods of Wan 2014. **Other adverse effects** Not fully reported and unclear how these were assessed; narrative description that "we did not have any complications" Data on ear pain are reported, but not reported separately for each group, only as the total number of study participants who experienced pain **Other outcomes reported in the study:** Subjective improvement in vertigo at 24 months follow‐up Speech discrimination scores Subjective improvement in hearing loss at 24 months (number of participants reporting 90% or 100% improvement) Aural fullness Electronystagmography	
Funding sources	Not reported	
Declarations of interest	Not reported	
Notes	**Research integrity checklist:** No retractions/expressions of concern No published protocol was identified, but this trial was published in 2005 Baseline characteristics do not appear to be excessively similar between the groups. Individual patient data are reported to assess this. Plausible loss to follow‐up occurred during the trial No implausible results were identified Equal numbers of participants (and equal proportions of males and females) were allocated to each group, without mention of blocked randomisation	
***Risk of bias***		
**Bias**	**Authors' judgement**	**Support for judgement**
Random sequence generation (selection bias)	Unclear risk	Quote: "Patients were randomised into 2 groups". Comment: no further details were provided regarding the process of randomisation.
Allocation concealment (selection bias)	Unclear risk	No details were provided regarding concealment of allocation, therefore we are unable to assess this domain.
Blinding of participants and personnel (performance bias) All outcomes	Low risk	Quote: "Neither patient nor physician knew the contents of the injection". Comment: likely that blinding of participants and personnel was adequate.
Blinding of outcome assessment (detection bias) All outcomes	Unclear risk	Comment: although the authors state that "neither patient nor physician knew the contents of the injection", participants who failed to respond to treatment at 6 months were able to have additional treatments if they were in the placebo arm. The point of un‐blinding is not described in the paper ‐ it is not clear how the physician would have determined that the participant could received another intervention. Therefore it is possible that outcomes may have been assessed with the knowledge of group allocation.
Incomplete outcome data (attrition bias) All outcomes	High risk	Comment: after 6 months, participants who received certain alternative interventions (in the placebo arm) were no longer followed up. This led to significant differences in attrition between the arms of the study, with only 7/11 participants completing full follow‐up in the placebo arm, compared to 11/11 in the intervention arm. 6‐month follow‐up is complete, therefore data at this time point can be considered low risk.
Selective reporting (reporting bias)	Unclear risk	Comment: no published protocol was identified to assess the possibility of selective reporting.
Other bias	Low risk	No other concerns identified.

**Table:** Lambert 2012

***Study characteristics***		
Methods	Parallel‐group, double‐blind RCT with single administration of intervention and follow‐up for 12 weeks	
Participants	**Setting:** Multicentre trial conducted at 15 sites in the USA from March 2010 to March 2011 **Sample size:** **Number randomised:** 44 participants **Number completed:** 44 participants **Participant baseline characteristics** **Age:** OTO‐104 3 mg group: median 53.0 years (range 26 to 60) OTO‐104 12 mg group: median 55.5 years (range 22 to 70) Placebo group: median 47.0 years (range 30 to 75) **Gender:** OTO‐104 3 mg group: 8 male (57%): 6 female (43%) OTO‐104 12 mg group: 7 male (44%): 9 female (56%) Placebo group: 5 male (36%): 9 female (64%) **Probable/definite Ménière's disease:** All definite Ménière's disease **Duration of disease:** Not reported **Attack frequency at baseline:** OTO‐104 3 mg group: mean 7.3 episodes per month (SD 5.98); proportion of days affected by vertigo: 0.261 (SD 0.2111) OTO‐104 12 mg group: mean 8.0 episodes per month (SD 5.43); proportion of days affected by vertigo: 0.290 (SD 0.1953) Placebo group: mean 8.4 episodes per month (SD 7.35); proportion of days affected by vertigo: 0.299 (SD 0.2599) **Hearing loss at baseline:** Not reported **Measure of tinnitus at baseline:** OTO‐104 3mg group: mean THI 52.5 (SD 25.76) OTO‐104 3mg group: mean THI 47.8 (SD 25.44) Placebo group: mean THI 58.4 (SD 29.50) **Number of participants with bilateral disease:** All participants had unilateral disease **Inclusion criteria:** Aged 18 to 75 years old. Unilateral Ménière's disease, according to the AAO‐HNS 1995 criteria. 2 or more episodes of vertigo per month for 2 months before the study. 2 or more episodes of vertigo during the 4‐week lead‐in period. Asymmetric, low‐frequency sensorineural hearing loss (a minimum of 25 dB decrement at 250 Hz in the affected ear). Disease duration less than 20 years. A history of using a low salt diet and/or diuretics for ≥ 1 month without relief of symptoms. **Exclusion criteria:** Infection in the ear, sinuses or upper respiratory system. History of immunodeficiency. Active or recent (within 1 month) middle ear disease. Abnormality of the tympanic membrane in the affected ear that would preclude intratympanic injection. History of endolymphatic sac surgery. Previous use of gentamicin in the affected ear. Use of systemic or intratympanic steroids within 1 month of screening. Pregnancy or lactation. History of tympanostomy tubes with evidence of perforation or lack of closure. Previous adverse reaction of IT injection of steroids. Use of an investigational drug or device in the 3 months prior to screening. **Diagnosis of Ménière's disease:** Diagnosed with definite, unilateral Ménière's disease according to the AAO‐HNS 1995 guidelines	
Interventions	**Intervention (n = 30 randomised, n = 30 completed)** A single injection of OTO‐104 was administered. This is a suspension of dexamethasone in a solution that includes a glycol polymer and forms a gel at body temperature. "A single 200 microlitre intratympanic injection was performed after application of phenol solution to the posterioinferior quadrant of the tympanic membrane, using a 3.5 inch 26 gauge spinal needle." The drug was injected near the round window niche. Either 3 mg or 12 mg of drug was administered. Data from both the 3 mg and 12 mg groups for OTO 104 have been pooled for analysis. **Comparator (n = 14 randomised, n = 14 completed)** The procedure was identical to that described above but using placebo (contents not stated) **Background interventions administered to all participants** None reported	
Outcomes	**Primary outcomes relevant to this review:** **Improvement in vertigo** Not reported **Change in vertigo** Change in the frequency of definitive vertigo episodes was assessed at 3 months. Participants completed a daily vertigo diary using the 'Gate's score', and a definitive vertigo episode was one lasting at least 20 minutes. If multiple attacks occurred on the same day, only the worst attack was reported. Vertigo frequency was calculated as the proportion of definitive vertigo days per month. **Serious adverse events** This was a phase 1b safety study; serious adverse effects were systematically assessed and reported **Secondary outcomes relevant to this review:** **Disease‐specific health‐related quality of life** This outcome was assessed using the MDPOSI questionnaire, but only a narrative summary of the outcome was reported **Hearing** Hearing was assessed as a component of the safety data for this study; a narrative summary of the outcome was reported **Tinnitus ** Assessed using the THI at 3 months **Other adverse effects** This was a phase 1b safety study; adverse effects were systematically collected and reported **Other outcomes reported in the study:** Systemic plasma dexamethasone levels Use of additional symptomatic relief medications	
Funding sources	The study was supported in full by Otonomy, Inc.	
Declarations of interest	"P.L. has received financial compensation from Otonomy, Inc., in the past but not related to his role as lead investigator in this study. S.N. is a current employee of Medical University of South Carolina. D.T. and L.L. were members of the Data Review Group and received financial compensation from Otonomy, Inc., for services performed on this safety committee. M.F. is currently an employee of INC Research, Inc., the Clinical Research Organization that was compensated by Otonomy, Inc.for services performed on this study. M.B. is a paid consultant to Otonomy, Inc. C.L. is currently an employee of Otonomy, Inc."	
Notes	**Research integrity checklist:** No retractions/expressions of concern Baseline characteristics of the groups are not excessively similar No loss to follow‐up occurred No implausible results were identified The numbers randomised to each group are compatible with an adequate randomisation process	
***Risk of bias***		
**Bias**	**Authors' judgement**	**Support for judgement**
Random sequence generation (selection bias)	Unclear risk	Quote: "After lead‐in, and starting with the OTO‐104 low‐dose cohort (3 mg), patients were randomly assigned to treatment with either OTO‐104 or placebo in a 2:1 ratio such that 14 patients received 3 mg of OTO‐104 and 7 patients received placebo." Comment: no further information is provided. We presume that random assignment to the high‐dose cohort was conducted in the same way.
Allocation concealment (selection bias)	Unclear risk	Quote: "patients were randomly assigned to treatment". Comment: no further details provided regarding allocation concealment.
Blinding of participants and personnel (performance bias) All outcomes	Low risk	Quote: "One milliliter syringes were loaded with appropriate study material by an unblinded pharmacist, with an accompanied label to obscure the appearance of the syringe contents." Comment: study stated to be triple‐blind to participants, personnel and outcome assessors.
Blinding of outcome assessment (detection bias) All outcomes	Low risk	Quote: "One milliliter syringes were loaded with appropriate study material by an unblinded pharmacist, with an accompanied label to obscure the appearance of the syringe contents." "To maintain the double‐blind, the physician left the examination room after completing the injection procedure, and a healthcare provider blinded to study drug entered the examination room and recorded patient‐reported post injection symptoms. The treating physicians were also excluded from administering study questionnaires that derived clinical activity associated with treatment and focused patient interactions exclusively on safety assessments." Comment: study stated to be triple‐blind to participants, personnel and outcome assessors.
Incomplete outcome data (attrition bias) All outcomes	Low risk	Comment: no missing outcome data.
Selective reporting (reporting bias)	Unclear risk	Comment: limited data are reported for hearing level and quality of life outcomes, precluding meta‐analysis.
Other bias	Low risk	Comment: no other concerns identified.

**Table:** Lambert 2016

***Study characteristics***		
Methods	Parallel‐group, double‐blind RCT with single dose of intervention and 4 months of follow‐up	
Participants	**Setting:** Multicentre study conducted at 15 sites in the USA; recruitment from December 2013 until April 2015 **Sample size:** **Number randomised:** 154 participants **Number completed:** 148 participants **Participant baseline characteristics** **Age:** OTO‐104 group: mean 54.8 (SD 11.04) years Placebo group: mean 55.3 (SD 12.48) years **Gender:** OTO‐104 group: 35 (45.5%) male: 42 (54.5%) female Placebo group: 39 (50.6%) male: 38 (49.4%) female **Probable/definite Ménière's disease:** All participants had definite disease **Duration of disease:** OTO‐104 group ≤ 5 years: 48 (62.3%) 6 to 10 years: 11 (14.3%) 11 to 15 years: 12 (15.6%) > 15 years: 6 (7.8%) Placebo group ≤ 5 years: 47 (61.0%) 6 to 10 years: 19 (24.7%) 11 to 15 years: 9 (11.7%) > 15 years: 2 (2.6%) **Attack frequency at baseline:** OTO‐104 group: proportion of days with definitive vertigo episode during the 1‐month lead‐in period: 0.282 Placebo group: proportion of days with definitive vertigo episode during the 1‐month lead‐in period: 0.252 **Hearing loss at baseline:** Not reported **Measure of tinnitus at baseline:** Not reported **Number of participants with bilateral disease:** All participants had unilateral disease. **Inclusion criteria:** Aged 18 to 85 years. Diagnosis of definite unilateral Ménière's disease, according to the AAO‐HNS 1995 guidelines. Asymmetric sensorineural hearing loss. At least 2 definitive vertigo episodes (a score of 2 to 4 from the vertigo severity scale) during the 4‐week lead‐in period. Completed at least 22 of 28 diary entries during screening. Patient agreed to maintain their current treatments for Ménière's disease; women of childbearing potential had a negative pregnancy test before randomisation and took adequate contraceptive precautions for the duration of the study. **Exclusion criteria:** Infection in the sinuses or upper respiratory system. Middle ear disease or a significant abnormality of the tympanic membrane affecting the IT injection. History of immunodeficiency disease. Previous use of IT gentamicin. Previous endolymphatic sac surgery. Tympanostomy tubes with evidence of perforation or lack of closure. Vertiginous migraine. Drop attacks. Systemic or IT steroids within 1 month prior to the trial. Experience of an adverse reaction to IT injection of steroids. Pregnancy or lactation. **Diagnosis of Ménière's disease:** Diagnosed with definite, unilateral Ménière's disease according to the AAO‐HNS 1995 guidelines	
Interventions	**Intervention (n = 77 randomised, n = 74 completed)** OTO‐104 (a suspension of dexamethasone in a polymer which gels at body temperature) was administered as a single 0.2 mL intratympanic injection of 60 mg/mL **Comparator (n = 77 randomised, n = 74 completed)** Placebo (contents not stated) was administered in an identical way to the active treatment **Background interventions administered to all participants** None reported; participants continued their baseline treatment throughout the study	
Outcomes	**Primary outcomes relevant to this review:** **Improvement in vertigo** Not reported **Change in vertigo** Vertigo severity was assessed with the 'Gate's score', using a daily diary; this score (range 0 to 4) captures the presence of vertigo, the duration of episodes and the severity of episodes Assessed with the change in frequency of definitive vertigo episodes and reported as the change in proportion of days during which a definitive episode occurred **Serious adverse events** Assessed and reported as treatment‐emergent adverse events **Secondary outcomes relevant to this review:** **Disease‐specific health‐related quality of life** No disease‐specific quality of life results are reported; results are only given for a generic quality of life questionnaire, the SF‐36 **Hearing** Not reported as continuous data; only reported as the number of participants who had an improvement of more than 10 dB in air bone gap at different frequencies (500 Hz, 1000 Hz and 2000 Hz) **Tinnitus ** Assessed using the THI, but data are only reported narratively **Other adverse effects** Some adverse events of relevance to this review were assessed and reported (tympanic membrane perforation, ear pain) **Other outcomes reported in the study:** Laboratory results Vital signs Columbia‐Suicide Rating Scale Word Recognition Tests Tympanometry	
Funding sources	The trial and analyses were financially supported by Otonomy, Inc.	
Declarations of interest	"P.L., J.C., and A.M. have received financial compensation from Otonomy, Inc., in the past but not related to their roles as investigators in this study. C.L. is currently an employee of Otonomy, Inc. and was involved the trial design and operation for the trial."	
Notes	**Research integrity checklist:** No retractions/expressions of concern Baseline characteristics of the 2 groups are not excessively similar Some loss to follow‐up was reported and reasons for this were described No implausible results were identified Computer‐generated block‐randomisation was used, which accounts for equal allocation of participants to the groups	
***Risk of bias***		
**Bias**	**Authors' judgement**	**Support for judgement**
Random sequence generation (selection bias)	Low risk	Quote: "eligible patients were randomly assigned to receive either 12 mg OTO‐104 or placebo on Day 1 using a 1:1 allocation ratio based on a computer‐generated permuted block randomization algorithm." Comment: adequate method of randomisation appears to have been used.
Allocation concealment (selection bias)	Low risk	Quote: "eligible patients were randomly assigned to receive either 12 mg OTO‐104 or placebo on Day 1 using a 1:1 allocation ratio based on a computer‐generated permuted block randomization algorithm." Comment: participants were allocated to groups using a computer‐generated block‐randomisation method, across multiple sites, indicating that group allocation would have been concealed.
Blinding of participants and personnel (performance bias) All outcomes	Low risk	Quote: " This was a [...] double‐blind, placebo‐controlled [...] study" "The administering physicians were trained not to use video monitors or discuss the appearance of the injected materials that would unblind the study staff or patients". Comment: study participants and personnel were apparently blinded to group assignment.
Blinding of outcome assessment (detection bias) All outcomes	Low risk	Comment: primary outcome measures were assessed by blinded participants.
Incomplete outcome data (attrition bias) All outcomes	Low risk	Comment: few dropouts, and these were balanced across the 2 groups.
Selective reporting (reporting bias)	High risk	Comment: trial registry record indicates that primary outcome measures would be reported at 4 months, whereas this is stated (and reported) at 3 months in the article. No 4‐month data are reported. Some outcomes are not reported in a way that does not allow for meta‐analysis, even though these data would have been collected (e.g. tinnitus data, some audiometric outcomes).
Other bias	Low risk	Comment: no other concerns identified.

**Table:** NCT02265393

***Study characteristics***		
Methods	Parallel‐group, double‐blind RCT with 6 months duration of treatment (2 injections). Followed by additional 6 months of open‐label treatment with the intervention for all participants in the trial. Data have only been extracted up to the 6‐month time point, which preserves the randomised comparison for this study. At later time points all participants had received the study drug.	
Participants	**Setting:** Multicentre study conducted at 16 sites in the UK; recruitment from July 2014 until May 2016 **Sample size:** **Number randomised:** 128 participants **Number completed:** 121 participants **Participant baseline characteristics** **Age:** OTO‐104 group: median 54 years (range 21 to 78) Placebo group: median 55 years (range 31 to 75) **Gender:** OTO‐104 group: 49 (48%) male: 54 (52%) female Placebo group: 9 (36%) male: 16 (64%) female **Probable/definite Ménière's disease:** Not reported **Duration of disease:** Not reported **Attack frequency at baseline:** Not reported **Hearing loss at baseline:** Not reported **Measure of tinnitus at baseline:** Not reported **Number of participants with bilateral disease:** All participants had unilateral disease **Inclusion criteria:** Diagnosis of unilateral Ménière's disease, according to the AAO‐HNS 1995 guidelines. Agrees to maintain their current standard of care treatments for Ménière's disease while on study. **Exclusion criteria:** History of immunodeficiency disease. Previous use of IT gentamicin. Previous endolymphatic sac surgery. Experience of an adverse reaction to IT injection of steroids. History of drop attacks. Pregnancy or lactation. **Diagnosis of Ménière's disease:** Diagnosed with unilateral Ménière's disease according to the AAO‐HNS 1995 guidelines	
Interventions	**Intervention (n = 103 randomised, n = 97 completed)** OTO‐104 (a suspension of dexamethasone in a polymer which gels at body temperature) was administered as a 0.2 mL intratympanic injection of 60 mg/mL. Two injections were given at 3‐monthly intervals, over the course of 6 months. **Comparator (n = 77 randomised, n = 74 completed)** Placebo (containing the inactive polymer only) was administered in an identical way to the active treatment, 2 injections over the course of 6 months **Background interventions administered to all participants** None reported. Participants continued their baseline treatment throughout the study.	
Outcomes	**Primary outcomes relevant to this review:** **Improvement in vertigo** Not reported **Change in vertigo** Vertigo severity was assessed with a Ménière's Symptom Questionnaire, with categories for vertigo, tinnitus, ear fullness and hearing difficulty. Results are only reported at 12 months (after all participants had received the active drug) therefore are not relevant to this review. **Serious adverse events** Assessed and reported at 6 months **Secondary outcomes relevant to this review:** **Disease‐specific health‐related quality of life** Not assessed or reported **Hearing** Only reported at 12 months, using a participant‐rated subjective score of hearing and using change from baseline in pure‐tone average. Results are only reported at 12 months (after all participants had received the active drug) therefore are not relevant to this review. **Tinnitus ** Assessed at 12 months using a symptom questionnaire, but this does not capture the impact of tinnitus on quality of life, and also does not compare the intervention to placebo (all participants had received active drug by this time point) **Other adverse effects** All adverse events of relevance to this review were assessed and reported at 6 months **Other outcomes reported in the study:** Tympanometry Aural fullness at 12 months	
Funding sources	The trial and analyses were financially supported by Otonomy, Inc.	
Declarations of interest	Results of this study are not published in a journal article, therefore there is no formal declaration of interest, but we note that the trial was sponsored and conducted by Otonomy Inc.	
Notes	**Research integrity checklist:** No retractions/expressions of concern have been identified, but the study is unpublished, so this is to be expected Baseline characteristics of the 2 groups are not excessively similar Some loss to follow‐up was reported and reasons for this were described No implausible results were identified Randomisation was apparently 4:1, but this is in keeping with a safety study	
***Risk of bias***		
**Bias**	**Authors' judgement**	**Support for judgement**
Random sequence generation (selection bias)	Low risk	Quote: "Central randomization system" Comment: method of randomisation is not explicitly stated, but is likely to be adequate.
Allocation concealment (selection bias)	Low risk	Quote: "Central randomization system" Comment: use of a central randomisation system is likely to ensure adequate concealment of group allocation.
Blinding of participants and personnel (performance bias) All outcomes	Low risk	Quote: "Study site personnel, who were blinded to treatment assignment" Comment: study site personnel and participants blinded to treatment allocation.
Blinding of outcome assessment (detection bias) All outcomes	Low risk	Comment: outcomes assessed by blinded study personnel or trial participants.
Incomplete outcome data (attrition bias) All outcomes	Low risk	Comment: few dropouts, likely to be insufficient to cause a risk of bias in the results.
Selective reporting (reporting bias)	Unclear risk	Comment: primary outcomes are reported according to the trial registration. It is unclear why efficacy outcomes (e.g. Ménière's Symptom Questionnaire) were not also reported at the 6‐month time point (allowing for comparison between the groups).
Other bias	Low risk	Comment: no other concerns identified.

**Table:** NCT03664674

***Study characteristics***		
Methods	Phase 3, parallel‐group RCT with a single dose of OTO‐104 and 3 months of follow‐up	
Participants	**Setting:** Multicentre trial based in USA and Europe (Belgium, Germany, Italy, Poland, Spain, Turkey and the UK) **Sample size:** **Number randomised:** 149 participants **Number completed:** not reported **Participant baseline characteristics** No characteristics are reported; assumed definite, unilateral Ménière's disease, due to inclusion criteria **Inclusion criteria:** Aged 18 to 85 years. Diagnosis of unilateral Meniere's disease by AAO‐HNS 1995 criteria and active vertigo for the 2 months prior to the study lead‐in period. Asymmetric sensorineural hearing loss. Agrees to maintain their current treatments for Ménière's disease while on‐study. **Exclusion criteria:** Pregnancy or lactation. History of immunodeficiency disease. History of previous endolymphatic sac surgery. History of previous use of intratympanic (IT) gentamicin in the affected ear. History of tympanostomy tubes with evidence of perforation or lack of closure. Previous adverse reaction to IT injection of steroids. Use of an investigational drug or device in the 3 months prior to screening. Previously been randomized to a trial of OTO‐104. **Diagnosis of Ménière's disease:** Diagnosed with Ménière's disease according to the AAO‐HNS 1995 guidelines. Presumed definite disease, as inclusion criteria state the need for documented sensorineural hearing loss.	
Interventions	**Intervention (number randomised and completed not reported)** Single intratympanic injection of 12 mg OTO‐104 **Comparator (number randomised and completed not reported)** Single intratympanic injection of placebo **Background interventions administered to all participants** Not reported	
Outcomes	**Primary outcomes relevant to this review:** **Improvement in vertigo** Not reported **Change in vertigo** Assessed with number of definitive vertigo days per month at 3 months; not fully reported **Serious adverse events** Assessed, according to trial registry site, but not reported **Secondary outcomes relevant to this review:** **Disease‐specific health‐related quality of life** Trial registry site states that "impact of vertigo experience on daily activities" will be assessed, but this is not reported **Hearing** Trial registry states that this will be assessed with audiometry at 3 months but no data are reported **Tinnitus ** Not apparently assessed or reported **Other adverse effects** Assessed, according to trial registry site, but not reported **Other outcomes reported in the study:** Otoscopic examinations, but these results are not reported	
Funding sources	Sponsored by Otonomy Inc.	
Declarations of interest	This trial remains unpublished, but was funded by Otonomy Inc.	
Notes	This trial was completed in December 2020. The results are currently unpublished, and no results are posted on the trial registry site. We have contacted the company, but they have been unable to supply us with the results of this trial. The only data available are published in a press release on the company website.	
***Risk of bias***		
**Bias**	**Authors' judgement**	**Support for judgement**
Random sequence generation (selection bias)	Unclear risk	Comment: this trial remains unpublished. No information is available to assess the risk of bias.
Allocation concealment (selection bias)	Unclear risk	Comment: this trial remains unpublished. No information is available to assess the risk of bias.
Blinding of participants and personnel (performance bias) All outcomes	Unclear risk	Comment: this trial remains unpublished. No information is available to assess the risk of bias.
Blinding of outcome assessment (detection bias) All outcomes	Unclear risk	Comment: this trial remains unpublished. No information is available to assess the risk of bias.
Incomplete outcome data (attrition bias) All outcomes	Unclear risk	Comment: this trial remains unpublished. No information is available to assess the risk of bias.
Selective reporting (reporting bias)	Unclear risk	Comment: this trial remains unpublished. No information is available to assess the risk of bias.
Other bias	Unclear risk	Comment: this trial remains unpublished. No information is available to assess the risk of bias.

**Table:** Ul Shamas 2017

***Study characteristics***		
Methods	Parallel‐group RCT. Presumed to be an unblinded study. Single administration of intervention followed by 6 months of follow‐up. Trial involved 3 arms: IT corticosteroids, IT gentamicin, IT placebo. For the purposes of this review only the arms considering IT corticosteroids and IT placebo have been reported.	
Participants	**Setting: ** Single‐centre trial conducted at a district hospital in India from February to August 2015 **Sample size: ** **Number randomised:** 40 participants **Number completed:** 40 participants **Participant baseline characteristics** **Age:** Not reported **Gender:** Not reported **Probable/definite Ménière's disease:** Not reported **Duration of disease:** Not reported **Attack frequency at baseline:** Not reported **Hearing loss at baseline:** IT corticosteroid group: mean pure tone average hearing threshold at speech frequencies: 42 (SD not reported) Placebo group: mean pure tone average hearing threshold at speech frequencies: 48 (SD not reported) **Measure of tinnitus at baseline:** IT corticosteroid group: mean THI 'grade' was 2.5 at baseline (SD not reported) Placebo group: mean THI 'grade' was 2.5 at baseline (SD not reported) It is unclear what is meant by the THI 'grade' **Number of participants with bilateral disease:** Not reported **Inclusion criteria:** Ménière's disease according to the criteria of the AAO‐HNS 1995. Persistent symptoms despite "maximal medical treatment" (this treatment is not described further in the paper). **Exclusion criteria:** None reported **Diagnosis of Ménière's disease:** AAO‐HNS 1995 criteria were used. Unclear if probable/definite disease.	
Interventions	**Intervention (n = 20 randomised, n = 20 completed)** Dexamethasone 0.5 mL (4 mg/mL) was administered (2 mg total) using a 27 G spinal needle and a 2 mL syringe. The drug was administered in "the anterograde inferior quadrant of the tympanic membrane", with the head tilted to the normal ear and maintained in position for 20 minutes. **Comparator (n = 20 randomised, n = 20 completed)** Intratympanic placebo (composition not stated) was administered in the same way as with the active drug **Background interventions administered to all participants** None reported	
Outcomes	**Primary outcomes relevant to this review:** **Improvement in vertigo** Not reported **Change in vertigo** Not reported **Serious adverse events** Not fully reported; some data on participants with profound hearing loss only **Secondary outcomes relevant to this review:** **Disease‐specific health‐related quality of life** Not reported **Hearing** Not reported in sufficient detail for inclusion in the review. Data are reported for the whole cohort of study participants, not for individual groups, therefore a comparison cannot be made between the groups. **Tinnitus ** THI 'grade' is reported for the individual groups, but the variance is not reported, therefore these data cannot be included in a meta‐analysis **Other adverse effects** Not reported fully ‐ no information on which groups participants were assigned to **Other outcomes reported in the study:** Nature of the tinnitus Hearing change across all cohort, not for individual groups	
Funding sources	Not reported	
Declarations of interest	Not reported	
Notes	**Research integrity checklist:** No retractions/expressions of concern No trial registration or protocol was identified Unable to assess baseline characteristics of the groups, as these are not reported fully Complete follow‐up was reported; no reasons for this were provided No implausible results were noted for this comparison (IT corticosteroids versus placebo/no treatment) Equal numbers of participants were allocated to each group, without mention of blocked randomisation	
***Risk of bias***		
**Bias**	**Authors' judgement**	**Support for judgement**
Random sequence generation (selection bias)	Unclear risk	Quote: "60 consecutive patients of Meniere’s disease were randomly divided into 3 groups of twenty patients each." Comment: very limited information regarding randomisation.
Allocation concealment (selection bias)	Unclear risk	Comment: no details regarding methods used to ensure group allocation was concealed.
Blinding of participants and personnel (performance bias) All outcomes	High risk	Comment: open‐label study. No blinding was used.
Blinding of outcome assessment (detection bias) All outcomes	High risk	Comment: open‐label study. No blinding of outcome assessors was reported.
Incomplete outcome data (attrition bias) All outcomes	Unclear risk	Comment: no details provided on attrition/exclusions from the study.
Selective reporting (reporting bias)	High risk	Comment: no published protocol or trial registration. Study does not apparently assess any vertigo outcomes, which would be considered key for Ménière's disease. Reporting of some outcomes was incomplete ‐ for example, the mean post‐treatment PTA for group B (intratympanic dexamethasone) was not reported; the rate of sensorineural hearing loss was not reported for groups B and C (intratympanic dexamethasone and saline respectively).
Other bias	High risk	Comment: very limited details provided regarding the nature and conduct of the study. Single‐author RCT with short period of recruitment for 60 participants in a single centre. The training and reliability of personnel assessing outcomes, and the validity of the instruments used, were not reported. No detail was provided on how many participants had unilateral/bilateral disease, and nor was the unit of analysis (person/ear) clarified. There was also a lack of data that could be used to compare outcomes across groups.

AAO‐HNS American Academy of Otolaryngology ‐ Head and Neck Surgery; DHI Dizziness Handicap Inventory; FLS Functional Level Scale; IQR interquartile range; IT intratympanic; MDPOSI: Meniere's Disease Patient Oriented Symptoms Index; PTA: pure tone average; RCT: randomised controlled trial; SF‐36 Short Form 36; SD: standard deviation; THI Tinnitus Handicap Inventory

### Characteristics of excluded studies [ordered by study ID]

**Table:**

Study	Reason for exclusion
Alles 2006	This is a systematic review. The reference list has been checked for studies that are potentially relevant for this review.
Conde 1965	This is a review article on the treatment of vertigo, not an RCT.
Dimitriadis 2017	This is a meta‐analysis and no new data are included. The reference list for this article has been checked to ensure any relevant RCTs are included in this review.
Godlowski 1965	This is a narrative review article and not an RCT.
Guo 2016	This is not an RCT.
Hao 2022	This is a meta‐analysis. The reference list has been checked to confirm that any eligible studies are included in this review.
Kitahara 2007	The intervention is not appropriate for this review: it includes surgery in addition to intratympanic steroids.
Kitahara 2008	The intervention is not appropriate for this review: it includes surgery in addition to intratympanic steroids.
Maksoud 2015	This is not an RCT.
NCT02768662	This is a non‐randomised safety study. It was due to provide long‐term safety data on 2 studies by Otonomy into OTO‐104. However, the trial was prematurely terminated.
Patel 2017	This is a narrative review and no new data are included. The reference list for this article has been checked to ensure any relevant RCTs are included in this review.
Phillips 2011	This is the previous Cochrane Review on this topic. The reference list has been checked to ensure any articles that meet the inclusion criteria for this review are included.
Richards 1971	This is a narrative review article and not an RCT.
Sakata 1988	This is not an RCT.
Silverstein 1998	This was a cross‐over trial. Although data are reported separately for the first phase of the trial, the duration of follow‐up (1 month) was insufficient to meet the inclusion criteria for this review (minimum of 3 months follow‐up required).
Syed 2015	This is a systematic review. The reference list has been checked to confirm that any relevant trials are included in this review.
Thabet 2008	This is not an RCT.

RCT: randomised controlled trial

## Differences between protocol and review

Due to the paucity of data for most outcomes, we were unable to carry out a number of pre‐planned sensitivity analyses and subgroup analyses. However, we added one unplanned sensitivity analysis in this review. As described in Sensitivity analysis, we assessed whether changing the planned outcome "improvement in vertigo" to "complete resolution or substantial improvement in vertigo" would alter the effect estimates. As this was a post hoc change, the results should be interpreted with caution. However, we considered that people with Ménière's disease may want to know if an intervention had a marked effect on their vertigo symptoms, rather than a more modest change. 

We planned to use the Trustworthiness Screening Tool from Cochrane Pregnancy and Childbirth to identify studies for inclusion in the main analysis. However, as described in Selection of studies, we did not exclude studies from the main analysis on the basis of concerns whilst using this tool. We considered that the overall certainty of the review findings (all very low‐ or low‐certainty) would not be impacted by this decision. 

As planned in our protocol, if the AAO‐HNS 1995 criteria had not been used to diagnose Ménière's disease, we considered whether to include the study. We took the post hoc decision that if a study had been conducted in an ENT department then it was reasonably likely that efforts had been made to exclude other possible diagnoses. This decision was taken by authors who were blinded to the other features and results of the studies. However, we acknowledge that this may result in a more heterogeneous population. We therefore reflected this by lowering the certainty of the evidence from studies with this limitation. 
